# Agent-Based Behavioral Modeling of Human Associative Learning in a Complex Approach-Avoidance Conflict Task

**DOI:** 10.1007/s42113-024-00229-1

**Published:** 2025-01-22

**Authors:** Franziska Usée, Sebastian Schmidt, Christiane A. Melzig, Dirk Ostwald

**Affiliations:** 1 Clinical Psychology, Experimental Psychopathology, and Psychotherapy, Philipps-Universität Marburg, Gutenbergstraße 29a, Marburg, 35032 Germany; 2 Center for Mind, Brain and Behavior, CMBB Philipps-Universität Marburg, Marburg, Germany; 3 Institute of Psychology, Otto von Guericke University, Universitätsplatz 2, Magdeburg, 39106 Germany

**Keywords:** Anxiety, Approach-avoidance behavior, Rescorla-Wagner learning, Agent-based behavioral modeling

## Abstract

Despite its key role in the development, maintenance, and treatment of anxiety disorders, the detailed mechanisms of human avoidance learning remain elusive. To contribute to the understanding of avoidance learning, we here report on a novel approach-avoidance conflict task that requires participants to learn associations between complex visual stimuli and combined appetitive and aversive stimuli while actively engaging with the experimental environment. Using an agent-based behavioral modeling approach, we implemented and validated an extensive set of control, heuristic, Rescorla-Wagner learning-based, and hybrid agents. We show that a Rescorla-Wagner learning-based agent with a prior expectation bias parameter best explains the learning behavior of 50 participants. As such, our work complements current research on the computational underpinnings of approach-avoidance behavior by showing paradigm and task instruction dependencies in approach-avoidance-relevant associative learning and contributes to the overall aim of achieving a more fine-grained understanding of the etiology of anxiety disorders.

## Introduction

From the earliest age, humans rely on their ability to learn associations between aversive experiences (e.g., getting stung by a wasp) and rather complex environmental contexts (e.g., characteristics of the place, season, weather, activity, movements), allowing them to avoid potentially (life-)threatening situations (Krypotos et al., 2015). However, in the absence of objective danger, learned avoidance responses no longer serve an adaptive role and may, in contrast, result in substantial psychological impairments. For example, socially anxious individuals may avoid making new friends or starting relationships to avoid the risk of being rejected. In this case, avoidance is costly and maladaptive, since potentially rewarding situations are forgone. Excessive avoidance behavior represents a core symptom of anxiety disorders, which are among the most common mental disorders worldwide (Barlow et al., 2002; World Health Organization, 2004; American Psychiatric Association, 2013; World Health Organization, 2017). Yet, despite its key role in theories on the development, maintenance, and treatment of anxiety disorders, the detailed mechanisms underlying human avoidance behavior remain elusive (Kirlic et al., 2017; Seriès, 2020; Hulsman et al., 2021).

In general, considering three experimental and methodological factors could contribute to a better understanding of avoidance behavior. First, most studies have thus far relied primarily on the use of overly simplistic and unambiguous stimuli typically associated with only appetitive (e.g., monetary gains) or aversive consequences (e.g., electrical shocks). However, in real life, maladaptive avoidance behavior is usually expressed in situations characterized by complex stimulus material and combined appetitive and aversive aspects (e.g., Lissek et al., 2006; Beckers et al., 2013). These situations are commonly referred to as *approach-avoidance conflicts* (AACs), with early psychological research on this topic dating back to the 1930s (Lewin, 1935).

Second, the design and implementation of translational AAC paradigms could contribute to the valid assessment of human avoidance learning and behavior (cf. Sierra-Mercado et al., 2015; Rolle et al., 2022). For experimental reasons, early research has focused mainly on non-human animal tests and human self-report measures (e.g., Carver and White, 1994; Kirlic et al., 2017). Moreover, many of the more recently developed paradigms use secondary reinforcers as punishment (e.g., loss of money or fictive points; Guitart-Masip et al., 2012; Pittig et al., 2015). However, in real life, punishment usually refers to primary threats (Aupperle et al., 2011), which limits the ecological validity of these experimental paradigms. Another limitation of the currently used AAC tasks concerns the intermingling of learning and decision-making processes. For example, many studies use multi-armed bandit paradigms, which require participants to decide between at least two options, each probabilistically associated with appetitive and/or aversive outcomes (e.g., Browning et al., 2015; Aylward et al., 2019; Moughrabi et al., 2022; Yamamori et al., 2023). Typically, these outcome probabilities are learned while making approach and avoidance decisions, which are rewarded or punished. Thus, participants are forced to actively learn while also optimizing their decisions for reward maximization and/or punishment minimization. As a consequence, learning and decision-making processes are difficult to dissociate experimentally, allowing no examination of pure associative learning and potential learning deficits. Other AAC tasks do not require any learning processes at all due to direct instruction or visualization of reward and punishment probabilities (Schrooten et al., 2014; Schlund et al., 2016; Zorowitz et al., 2019; Smith et al., 2021; Pedersen et al., 2021), which again limits the ecological validity of these studies (see also *description-experience gap*; Hertwig and Erev, 2009).

Third, despite the current renewed interest in investigating AACs (e.g., Sierra-Mercado et al., 2015; Meulders et al., 2016; Bublatzky et al., 2017; Kirlic et al., 2017; Bach et al., 2020; Moughrabi et al., 2022; Garcia-Guerrero et al., 2023), computational modeling approaches that bear the potential to dissect the fine-grained psychological mechanisms of avoidance learning and behavior are only beginning to be employed in clinical psychology and psychiatry (Mkrtchian et al., 2017; Zhang et al., 2020; Smith et al., 2021; Hunter et al., 2022; Moughrabi et al., 2022; Yamamori et al., 2023; Yamamori and Robinson, 2023). In contrast to classical statistical methods, computational modeling aims to explain trial-by-trial variations in observed behavior by explicitly and mathematically formulating, implementing, and testing theories of the underlying mechanisms (Montague et al., 2012; Friston et al., 2014; Seriès, 2020; Zhang et al., 2020; Paulus and Thompson, 2021). In the context of AACs and fear learning in general, Rescorla-Wagner learning-based models are particularly prominent (Rescorla and Wagner, 1972; Yau and McNally, 2023; Yamamori and Robinson, 2023).

To contribute to a better understanding of avoidance learning and motivated by the identified gaps in the literature, we here report on a novel approach-avoidance conflict task that requires participants to learn associations between complex visual stimuli and combined appetitive (i.e., fictive points) and aversive stimuli (i.e., electrotactile stimulation) while actively engaging with the experimental environment (cf. *visual foraging;* Kristjánsson et al., 2020). Notably, the development of the paradigm was inspired by non-human active exploration paradigms (Kirlic et al., 2017). In contrast to previous paradigms as reviewed above, our overall task design allows us to experimentally disentangle associative learning processes from approach-avoidance decisions and, using an agent-based behavioral modeling approach, to provide mechanistic insights into the associative learning mechanisms in approach-avoidance settings. To this end, we explore the potential of various Rescorla-Wagner learning-based agents for learning associations of complex visual stimuli and both rewards and punishment. Following the finding that separate learning rates for rewards and punishment are useful for explaining approach-avoidance behavior (Mkrtchian et al., 2017; Aylward et al., 2019; Yamamori et al., 2023), we compare agents with single or reward/punishment-specific learning rates. In addition, inspired by recent arguments on the optimality of trial-adaptive learning rates (Gershman, 2015), we also explore these types of agent models, given the strong inferential character of our task. Finally, using a decision value maximization approach as common in decision theory (Puterman, 2005; Dayan and Daw, 2008; Kochenderfer et al., 2022) allows us to also include control agents that explain actions without recourse to learning and to explore the potential of explaining task behavior with more parsimonious agents than the standard Rescorla-Wagner model (Wilson and Collins, 2019).

In general, our work is situated at the intersection of associative learning, approach-avoidance conflict, and computational modeling. The main contributions of our work are threefold. First, we focus exclusively on the acquisition of approach-avoidance behavior within a complex visual environment associated with both rewarding and threatening stimuli. Second, we explore Rescorla-Wagner learning-based strategies for complex inference decisions based on their recent success in the AAC literature. Third, we complement and deepen the current interest into simultaneous learning and approach-avoidance behavior by showing paradigm and task instruction dependencies of the learning phase. In the following, we first introduce the paradigm (“Experimental Methods” section) and continue with the descriptive analyses of experimentally observed learning behavior (“Descriptive Analyses” section). We then provide a detailed description and validation of our agent-based behavioral modeling framework, followed by the results of the model comparison when applied to the experimental data (“Agent-Based Behavioral Modeling” section). We close by discussing the practical implications of our findings and shortcomings of our approach (“Discussion” section). For the data and implementational details of this work, please refer to https://osf.io/bjdse/.

## Experimental Methods

### Participants

Participants were recruited through the Philipps-Universität Marburg research participant system, distribution lists, and social media platforms. In total, 54 participants gave their written informed consent for participation and dissemination of their anonymized data for research purposes. Due to technical issues (n=3) and premature termination of the experiment (n=1), four participants were excluded from the analysis, resulting in a final sample size of n=50 (66% female, 34% male). The sample consisted mainly of students (94%; 6% employees) between 18 and 33 years (M=22.98 years, SD=3.62). Most of the participants named a general qualification for university entrance as their highest level of education (82%; 16% with university degree; 2% with apprenticeship). The study was approved by the ethics committee of the Department of Psychology of Philipps-Universität Marburg (2022/04/07, processing sign: 2022-14v). Participants were informed that participation in the study is voluntary and may be terminated at any time without giving any reason. Participants received course credit or participated without any compensation.

### Procedure

The study consisted of a one-time visit of approximately two hours to the Philipps-Universität Marburg. After providing their written informed consent, participants were first asked to provide information on sociodemographic variables (sex, age, educational level, marital status, employment status) and to complete a series of questionnaires measuring anxiety- and mood-related constructs (Appendix A). The participants then received detailed written and oral instructions about the experimental paradigm and were encouraged to ask questions (Appendix B). To facilitate the understanding of the task, participants were also presented with a short video demonstrating the completion of exemplary trials (Appendix B). Subsequently, a bipolar electrode was attached to the participant’s left forearm for delivery of electrotactile stimulation. The intensity of electrotactile stimulation was individually adjusted using a standard procedure to ensure that the stimulation was subjectively perceived as “highly annoying but not painful” (Hamm and Vaitl, 1996; Krause et al., 2018).

Participants were appropriately seated in front of a computer monitor (approximately 60 cm distance; 24-inch monitor with a resolution of 1920 × 1080 pixels and a refresh rate of 120 Hz). To familiarize the participants with the task, they completed four practice trials on visual stimuli similar but not identical to those used in the main phases of the experiment. If task instructions were not followed or not fully met, participants were informed and relevant instructions were repeated verbally. Before starting with the first experimental phase, a standard 9-point eye movement recording calibration was performed. Finally, participants completed the three main phases of the experiment, while behavioral and eye movement data were recorded. Each phase of the experiment started with a summary of the task instructions relevant to the respective phase (Appendix B). After each experimental phase, participants were asked to answer several task-related questions on a tablet computer (Appendix A). The eye movement recording calibration procedure was repeated before entering the next experimental phase. After all experimental phases were completed, the stimulation electrode was removed and the participants were again asked to answer task- and mood-related questions (Appendix A). Note that in the current work, we only report results from the analysis of the behavioral data, but not the eye movement data.

### Electrotactile Stimulation

Electrotactile stimulation was applied using the Digitimer High Voltage Stimulator model DS7A. For individual adjustment of the electrotactile stimulation intensity, participants were first given mild electrotactile stimulation (1 mA, 2 ms pulse duration) which was then gradually increased until it was perceived as “highly annoying but not painful.” The average electrotactile stimulation intensity across participants was 5.93 mA (SD=5.21).

**Fig. 1:**
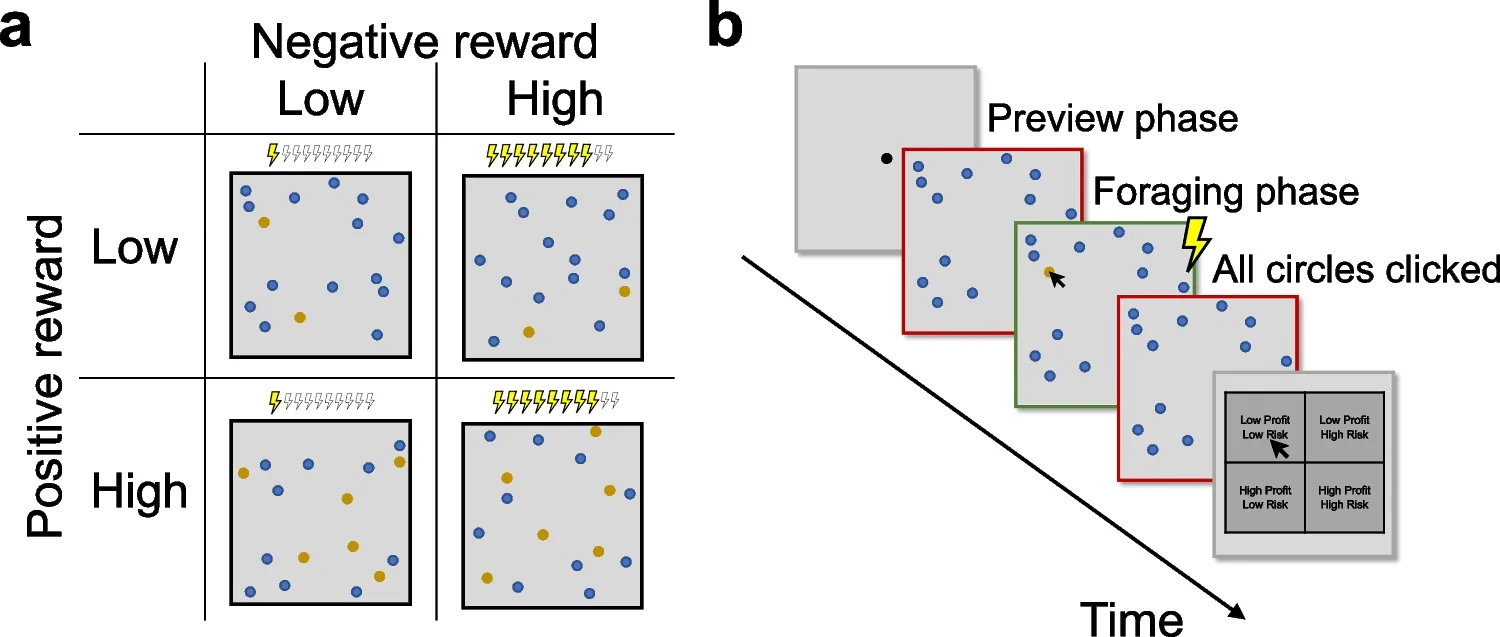
Approach-avoidance conflict paradigm. **a** Experimental design. Each visual search field pattern represented one of four experimental conditions. The experimental conditions were specified in terms of the number of hidden coins (*low* and *high* levels of the *positive reward* factor) and the probability of receiving electrotactile stimulation (*low* and *high* levels of the *negative reward* factor). **b** Visualization of the learning phase trial design. In each learning phase trial, participants were required to click on each blue circle at least once while simultaneously being at risk of electrotactile stimulation. Once all circles were clicked at least once, participants were forwarded to a contingency rating that served as a behavioral read-out of the learning process

### Experimental Design

We designed an approach-avoidance conflict paradigm, in which participants performed a foraging task while simultaneously being at risk of receiving electrotactile stimulation (cf. Schmidt et al., in preparation). The paradigm consisted of three experimental phases. In each phase, participants were repeatedly presented with four different visual search fields. Each of the four visual search fields consisted of 16 blue circles arranged in a unique spatial pattern. By clicking on each circle, participants could uncover golden coins, contributing to their fictive experimental score. Each of the four visual search fields represented one of four experimental conditions, constituting one of four cells of a 2 × 2 within-participants factorial design (Fig. 1a). Specifically, we manipulated the experimental factors *reward* and *punishment* (cf. Corr, 2013; Kirlic et al., 2017), which we here refer to as *positive reward* and *negative reward* in line with the standard reinforcement learning terminology (Sutton and Barto, 2018). The levels of the *positive reward* factor were *low*, corresponding to two hidden coins beneath the 16 blue circles of the visual search field, and *high*, corresponding to six hidden coins beneath the 16 blue circles of the visual search field. The levels of the *negative reward* factor were *low*, corresponding to a probability of 0.1 for electrotactile stimulation while searching a visual search field for golden coins, and *high*, corresponding to a probability of 0.8 for electrotactile stimulation while searching a visual search field for golden coins.

In the first phase of the experiment, hereinafter referred to as the *learning phase*, participants were asked to learn the associations between the visual search field patterns and the experimental conditions. Each visual search field was presented 10 times, resulting in a total of 40 learning phase trials. In each of these trials (Fig. 1b), participants were required to click on each blue circle in the presented search field at least once before being able to proceed to the next trial. After each trial, participants were asked to indicate the experimental condition associated with the visual search field by clicking on one of four options presented in a table-like format. As by design each visual search field pattern was associated with a specific *positive reward* and *negative reward* level combination, we refer to an experimental condition and the selection of a given option as a *reward state* and a *contingency rating*, respectively. In the second phase of the experiment, participants were again presented with the same four visual search fields in a total of 40 trials. In contrast to the learning phase, participants were required to decide for themselves whether and for how long they wanted to search for coins in each trial. Specifically, participants could only proceed to the next trial by actively clicking on an *Exit* button. In this phase, no contingency ratings were collected. In the third phase of the experiment, participants were again required to click on the Exit button to proceed to the next trial. This phase consisted of 12 trials, each visual search field being presented three times. Unlike the previous two experimental phases, participants were not at risk for electrotactile stimulation and were explicitly informed of its absence in this phase. Again, no contingency ratings were collected.

The assignment of visual search field patterns to reward states was counterbalanced across participants. The trial-by-trial sequence of visual search fields was pseudo-randomized in blocks of four trials, such that each visual search field occurred once in each block and the same visual search field could not be presented on two successive trials. The location of hidden coins changed on a trial-by-trial level and was randomly determined at the beginning of each experimental phase. That is, participants were not required to learn the exact locations of the coins but only the total amount of coins associated with a certain visual search field. The participants were aware that the number of coins collected was irrelevant to their reimbursement. The experimental trials on which electrotactile stimulation was applied were randomly determined for each participant and phase of the experiment. As a veridical representation of the probabilities of the low and high *negative reward* factor levels, participants received electrotactile stimulation in 1 of 10 trials of the low negative reward condition and in 8 of 10 trials of the high negative reward condition. In general, electrotactile stimulation was applied while participants actively engaged with the experimental environment. In each trial, participants could receive electrotactile stimulation up to two times. To prevent participants from feeling completely safe after the first electrotactile stimulation, participants were not informed about the number of trials in which electrotactile stimulation could occur twice. Specifically, 2 of 10 trials of the high negative reward condition were randomly determined to contain electrotactile stimulation twice. In the low negative reward condition, only a single electrotactile stimulation could occur in 1 of 10 trials.

The experimental task was implemented in Python 2.7 using OpenSesame 3.3.9 (Mathôt et al., 2012). In the present work, we develop computational models for and report behavioral results from the learning phase only. In the following, we provide further details on the trial design of this phase.

### Learning Phase Trial Design

Each learning phase trial started with the presentation of a fixation point for a duration of two seconds (Fig. 1b). Afterwards, the visual search field was presented in a surrounding red frame, indicating that participants could not yet search for coins, as the mouse cursor movement was deactivated. After another three seconds, the visual search field’s frame color changed to green, signaling the start of the foraging task. Specifically, participants were then able to search for coins by clicking on the blue circles while being at risk of receiving electrotactile stimulation. With respect to the positive reward component, the color of the respective circle changed to red in response to a mouse click, signaling the absence of a hidden coin, or gold, signaling the presence of a hidden coin. To collect a discovered coin, participants had to execute one further mouse click on the respective circle. Each coin collected increased the participant’s total score presented above the visual search field by one point. Each hidden coin could be collected only once. That is, if the blue circle was clicked again after collecting the coin, the circle’s color changed to red and not gold. With respect to the negative reward component, electrotactile stimulation could not occur while clicking on blue circles or golden coins to prevent participants from associating specific circles or clicks with negative reward. On trials with a single electrotactile stimulation, the electrotactile stimulus was then applied uniformly at random between the fourth and fifteenth mouse clicks. On trials with two electrotactile stimulation occurrences, the first electrotactile stimulus was applied uniformly at random between the fourth and eighth mouse clicks, while the second electrotactile stimulus was applied uniformly random between the tenth and fifteenth mouse clicks. Finally, after each blue circle was clicked at least once, the visual search field’s frame color changed back to red, signaling the end of the foraging task and the deactivation of mouse movements. After two seconds, participants were automatically forwarded to the contingency rating, asking them to indicate the reward state associated with the visual search field pattern just presented by clicking on one of the four cells in a table. There was no time limit for the contingency rating. Once participants provided their responses to the contingency rating, they continued with the next trial.

## Descriptive Analyses

Participants’ behavioral performance was assessed in terms of their trialwise responses to the contingency ratings, which we hereafter refer to as *actions*. At the descriptive level, we evaluated average action accuracies across participants as functions of reward states and learning phase trials. In addition, we report the associated standard errors of the mean (*SEMs*). To this end, we defined the action of a participant as *correct*, if the selected action corresponded to the experimentally presented reward state. Note that since participants were required to respond to each contingency rating to continue with the next trial, the experimental design did not allow missing actions.

**Fig. 2:**
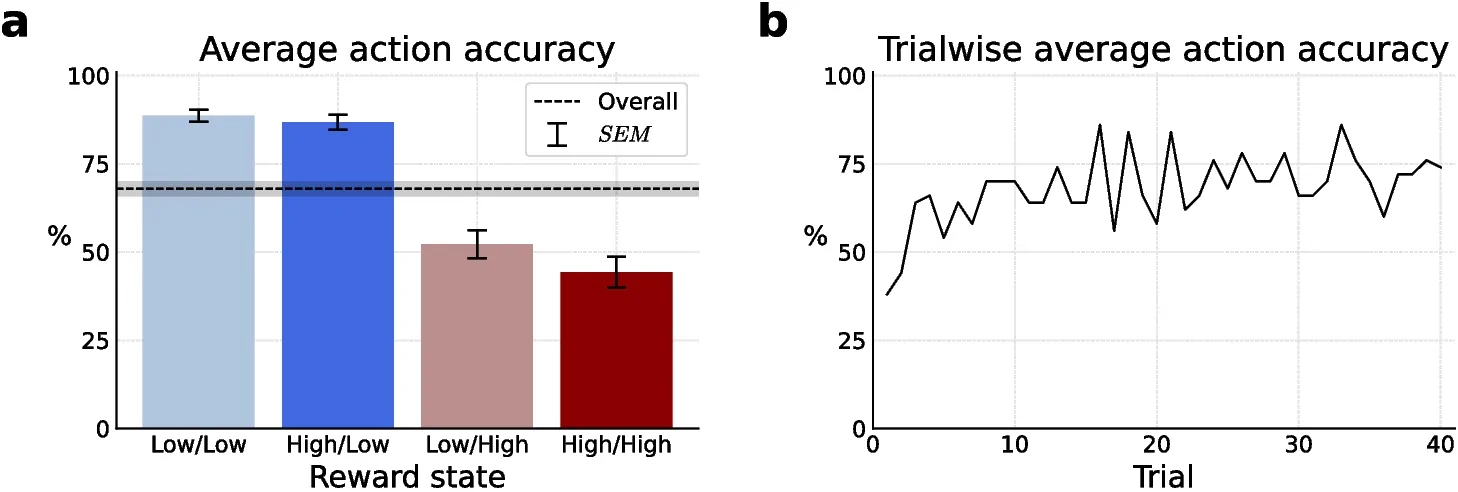
Average action accuracies. **a** Average action accuracy by reward state. The dashed line represents the overall average action accuracy across all reward states. The light gray area around this dashed line refers to the corresponding standard error of the mean (*SEM*). The colored bars represent the reward state-specific average action accuracies. Reward states are labeled according to the following principle: *positive reward level*/*negative reward level*. From left to right, the order of reward states is thus as follows: (1) low positive reward/low negative reward, (2) high positive reward/low negative reward, (3) low positive reward/high negative reward, (4) high positive reward/high negative reward. The error bars depict the *SEM*. **b** Trialwise average action accuracy across all reward states. For the generation of this figure, please see *abm_figure_2.py*

As shown in Fig. 2a, participants achieved an average action accuracy of 67.95% ± 2.1 across all reward states and trials. Notably, participants performed better for low negative reward states than for high negative reward states. Specifically, the highest average action accuracy of 88.6% ± 1.77 was observed for the reward state *low positive reward/low negative reward* and the lowest average action accuracy of 44.2% ± 4.39 was observed for the reward state *high positive reward/high negative reward*. For the reward states *high positive reward/low negative reward* and *low positive reward/high negative reward*, the action accuracy averaged to 86.8% ± 2.14 and 52.2% ± 3.96, respectively. As shown in Fig. 2b, the average action accuracy increased over trials from around 38 to 74%, indicating the successive acquisition of the correct associations. To assess the statistical significance of this increase in action accuracy, we conducted two analyses in the context of the general linear model. First, we parametrically evaluated the slope of the group data action accuracy using a simple linear regression analysis with trial number as independent variable and group average action accuracy as dependent variable using the Python *statsmodels* package (Seabold and Perktold, 2010). This analysis yielded a slope estimate of 0.43 action accuracy units per trial with an associated standard error estimate of 0.12, resulting in t(38)=3.57,p<.001, thus indicating statistically significant evidence against the null hypothesis of a slope parameter of zero, corresponding to no learning. Second, we categorically evaluated the participant-specific differences in the average action accuracy on the first three visual search field presentations of each condition (trials 1–12) vs. the average action accuracy on the last three visual search field presentations of each condition (trials 29–40) using a one-sample *t*-test as implemented in the Python *scipy* package (Virtanen et al., 2020). This test resulted in t(49)=4.9,p<.001, again indicating statistically significant evidence against the null hypothesis of no action accuracy change during the learning phase.

**Fig. 3:**
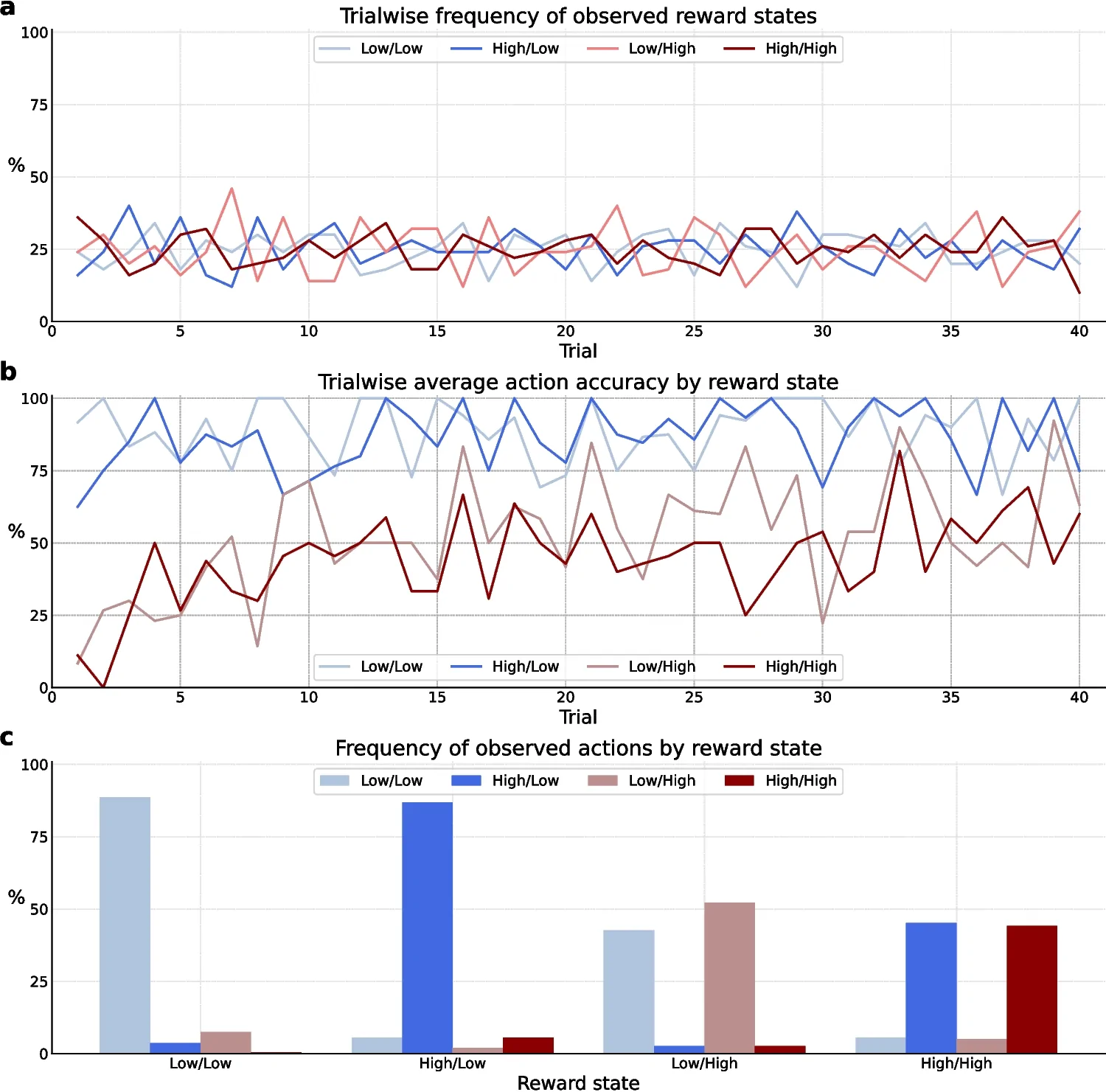
Reward state-specific evaluation of participants’ behavior. **a** Trialwise frequency of observed reward states. The participant-specific trial-by-trial sequence of reward states was pseudo-randomized in blocks of four trials, such that each reward state occurred once in each block and the same reward state could not be presented on two successive trials. For a given trial, the frequencies sum to 100% across observed reward states. The colors depict the reward states (*positive reward level*/*negative reward level*). **b** Trialwise average action accuracy by reward state. The colors depict the reward states (*positive reward level*/*negative reward level*). **c** Frequency of observed actions by reward state. The colored bars depict the frequency of participants’ actions for a given reward state. For a given reward state, the action frequencies sum to 100%. For the generation of this figure, please see *abm_figure_3.py*

To obtain further insight into the choice behavior underlying these globally observed patterns, we evaluated the reward state-specific trialwise average action accuracy and the reward state-specific action choice frequency (Fig. 3). We first note that there were no systematic differences in the trialwise frequency of the experimentally presented reward states (Fig. 3a). Yet, the initial performance of the participants differed strongly between the low and high negative reward states (Fig. 3b). As detailed in Fig. 3c, participants often falsely rated high negative reward states as low negative reward states, while low negative reward states and the level of positive reward were usually judged correctly. Together, participants thus often seemed to underestimate the presence of a negative reward.

## Agent-Based Behavioral Modeling

**Fig. 4:**
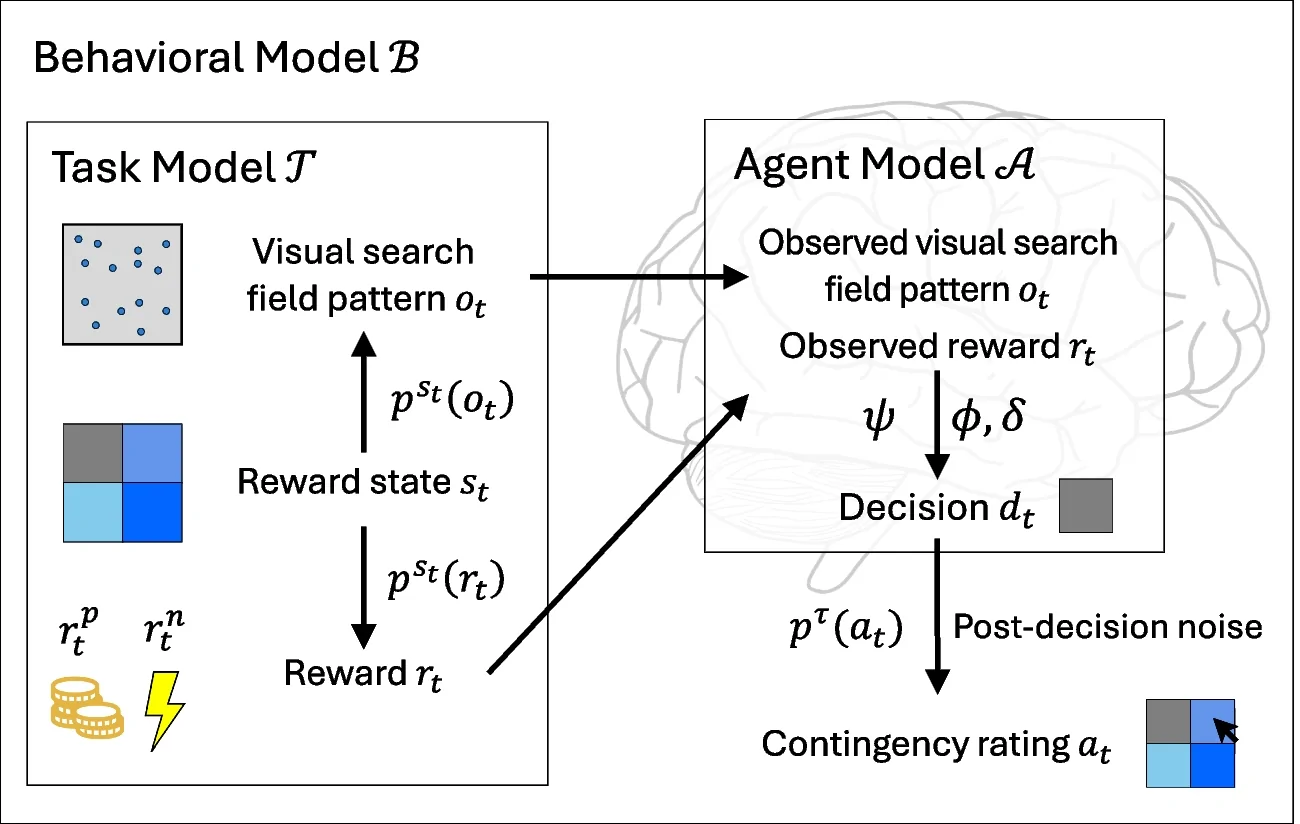
Conceptual overview of the agent-based behavioral modeling framework. In general, our agent-based behavioral modeling framework differentiates between a task model $\mathcal {T}$, an agent model $\mathcal {A}$, and their joint embedding in a behavioral model $\mathcal {B}$. The task model specifies key aspects and the structure of the experimental task. In the present work, the task model includes a trial-specific reward state $s_t$, a trial-specific observable visual search field pattern $o_t$, and a trial-specific reward as defined by a positive ($r_t^p$, that is, coins) and negative reward component ($r_t^n$, that is, electrotactile stimulation). The probability of a specific reward $r_t$ for a given reward state $s_t$ is defined by the reward state-dependent reward distribution $p^{s_t}(r_t)$. The probability of a specific visual search field pattern $o_t$ for a given reward state $s_t$ is defined by the reward state-dependent visual search field pattern observation distribution $p^{s_t}(o_t)$. The agent model encodes the subjective representation of the task components and several decision-making processes. To this end, the agent model has limited access to aspects of the task model. In the current study, access is limited to the observable search field patterns and rewards such that the true reward state $s_t$ remains unknown to the agent. For most of our agents, the learning and decision-making processes are represented by a learning function ψ that yields trial-by-trial expected reward value estimates, a decision value function ϕ that assigns values to each possible decision based on a predefined choice strategy and/or expected reward value estimate, and a decision function δ that selects a decision $d_t$ based on a predefined choice strategy and/or the decision values. Augmenting the interaction of task and agent model with an action probability distribution then provides a probabilistic embedding that allows for the transformation of latent agent decisions $d_t$ into observable actions $a_t$ under the influence of post-decision noise and renders the task-agent interaction amenable to statistical inference. We use the term *behavioral model* to refer to the joint hierarchy of task model, agent model, and their probabilistic augmentation

To arbitrate between different learning and decision-making strategies that participants may have used in the learning phase of the experiment, we employed an agent-based behavioral modeling approach akin to Horvath et al. (2021). This approach comprises a *task model*, a set of *agent models*, and their joint probabilistic embedding in *behavioral models*. We detail this model hierarchy in the “Model Formulation” section. In general, the task model captures key aspects of the learning phase task and encodes the agents’ knowledge about the learning environment or, equivalently, the participants’ knowledge about the learning phase task structure. The agent models then specify a set of subjective representations of the learning phase task and several decision-making processes based on these representations. For example, some of our agents compute trial-by-trial visual search field pattern-dependent expected reward value estimates. These trialwise expected reward value estimates are then used by the agents to decide on the latent reward state. Finally, the agents’ decisions are probabilistically embedded, such that they can be transformed into post-decision noise-afflicted actions, which in turn serve to model the experimentally observed participants’ contingency ratings. Accordingly, from a data-generative perspective, the probabilistic embedding of task and agent models allows for generating trial-by-trial contingency ratings as actions, while from a data-analytical perspective, it allows for statistically comparing the relative plausibilities of the agent models in light of simulated or experimental behavioral data. Figure 4 illustrates the agent-based behavioral modeling framework applied in the present work. Note that the agents serve as generative models of the between-trial learning process and thus only model the self-selected participants’ contingency ratings as actions, but not the mandatory within-trial sequence of mouse clicks on all blue circles.

Having formulated our modeling approach in the “ModelFormulation” section, we then continue with the documentation of our model parameter estimation and model comparison methods in the “Parameter Estimation and ModelComparison” section. Finally, we report the results of several model validation analyses (“Model Validation” section) and conclude with the evaluation of the agent-based behavioral models in light of the participants’ experimental data (“Evaluation of the Experimental Data” section). For our notational conventions, please refer to Appendix C.

### Model Formulation

#### Task Model

To allow for the simulation of task and agent interactions and to represent the agents’ and participants’ partial knowledge of the learning phase task structure, we first formulate a task model. Intuitively, this task model represents the core aspects of the learning phase of the experiment, as documented in the “Experimental Methods” section. Formally, the task model is given by the tuple

1 $$\begin{aligned} \mathcal {T} := (T,S,O,R,f,p^{s_t}(o_t),p^{s_t}(r_t)). \end{aligned}$$

Here,

- T:=40 denotes the number of trials, indexed by t=1,2,...,T.
- $S:= \{0,1,2,3\}$ denotes the set of reward states *s*, encoded as

∘: 0:= low positive reward, low negative reward,
∘: 1:= high positive reward, low negative reward,
∘: 2:= low positive reward, high negative reward, and
∘: 3:= high positive reward, high negative reward.

- $O:= \{0,1,2,3\}$ denotes the set of visual search field patterns *o* (cf. Appendix D).
- $$R:= \left\{ \left( \begin{array}{l} r^p \\ r^n \end{array}\right) | r^p, r^n \in \{-1,+1\}\right\} $$ denotes the set of rewards *r*, where $r^p$ encodes the *positive reward component* as

∘: -1:= low number of coins,
∘: +1:= high number of coins,

and $r^n$ encodes the *negative reward component* as

∘: -1:= presence of electrotactile stimulation,
∘: +1:= absence of electrotactile stimulation.

Note that we encode the observable positive and negative reward components in a categorical manner rather than in a parametric manner. Specifically, this means that rather than encoding the number of coins observed by their parametric values of 2 or 6, we categorically label them -1 and +1, respectively. Intuitively, this involves the assumption that agents distinguish between a low and a high positive reward state rather than a reward state of exactly 2 (but not, for example, 3) and exactly 6 (but not, for example, 7). Similarly, we encode the presence and absence of electrotactile stimulation categorically rather than by the exact number of electrical shocks. As detailed in the “Experimental Methods” section, for high negative reward states, the presence of electrotactile stimulation was operationalized by two electrical shocks in a small subset of trials. Finally, note that we encode the reward components in line with a net reward observation intuition. Specifically, the observation of a low number of coins and the presence of electrotactile stimulation results in a net reward of $r^p + r^n = -2$, the observation of a low number of coins and the absence of electrotactile stimulation results in a net reward of $r^p + r^n = 0$, the presence of a high number of coins and the presence of electrotactile stimulation positive reward results in a net reward of $r^p + r^n = 0$, and the presence of a high number of coins and the absence of electrotactile stimulation results in a net reward of $r^p + r^n = 2$.

- $f: \mathbb {N}_{T} \rightarrow S, t \mapsto f(t):= s_t$ denotes the reward state evolution function, which specifies the trial-specific reward state $s_t$. In particular, *f* corresponds to the participant-specific sequence of reward states as presented during data acquisition and retrieved from the behavioral data sets for simulations and analyses, which comprised 10 repetitions of each of the four reward states (cf. “Experimental Methods” section).
- $p^{s_t}(o_t)$ denotes the reward state-dependent visual search field pattern distribution, which is defined according to the deterministic association of the visual search field patterns with the reward states (cf. “Experimental Design” section) and takes the form
  2 $$\begin{aligned} p^{s_t = s}(o_t = o) := {\left\{ \begin{array}{ll} 1 & (o = s) \\ 0 & (o \ne s) \end{array}\right. } \text { for all } o \in O \text { and } s \in S. \end{aligned}$$
- $p^{s_t}(r_t)$ denotes the reward state-dependent reward distribution, which is defined according to the semi-determinis-tic association of the positive and negative reward components with the reward states (cf. “Experimental Design” section) and takes the form
  3 $$\begin{aligned} {\begin{matrix} & p^{s_t = 0}\left( r_t = \left( \begin{array}{l} -1 \\ -1 \end{array}\right) \right) := 0.1, \quad p^{s_t = 0}\left( r_t = \left( \begin{array}{l} +1 \\ -1 \end{array}\right) \right) := 0.0, \\ & p^{s_t = 0}\left( r_t = \left( \begin{array}{l} -1 \\ +1 \end{array}\right) \right) := 0.9, \quad p^{s_t = 0}\left( r_t = \left( \begin{array}{l} +1 \\ +1 \end{array}\right) \right) := 0.0, \\ & p^{s_t = 1}\left( r_t = \left( \begin{array}{l} -1 \\ -1 \end{array}\right) \right) := 0.0, \quad p^{s_t = 1}\left( r_t = \left( \begin{array}{l} +1 \\ -1 \end{array}\right) \right) := 0.1, \\ & p^{s_t = 1}\left( r_t = \left( \begin{array}{l} -1 \\ +1 \end{array}\right) \right) := 0.0, \quad p^{s_t = 1}\left( r_t = \left( \begin{array}{l} +1 \\ +1 \end{array}\right) \right) := 0.9, \\ & p^{s_t = 2}\left( r_t = \left( \begin{array}{l} -1 \\ -1 \end{array}\right) \right) := 0.8, \quad p^{s_t = 2}\left( r_t = \left( \begin{array}{l} +1 \\ -1 \end{array}\right) \right) := 0.0, \\ & p^{s_t = 2}\left( r_t = \left( \begin{array}{l} -1 \\ +1 \end{array}\right) \right) := 0.2, \quad p^{s_t = 2}\left( r_t = \left( \begin{array}{l} +1 \\ +1 \end{array}\right) \right) := 0.0, \\ & p^{s_t = 3}\left( r_t = \left( \begin{array}{l} -1 \\ -1 \end{array}\right) \right) := 0.0, \quad p^{s_t = 3}\left( r_t = \left( \begin{array}{l} +1 \\ -1 \end{array}\right) \right) := 0.8, \\ & p^{s_t = 3}\left( r_t = \left( \begin{array}{l} -1 \\ +1 \end{array}\right) \right) := 0.0, \quad p^{s_t = 3}\left( r_t = \left( \begin{array}{l} +1 \\ +1 \end{array}\right) \right) := 0.2. \\ \end{matrix}} \end{aligned}$$
  As an example of the logic underlying the definition of $p^{s_t}(r_t)$, consider the case of reward state $s_t = 2$ (low positive reward, high negative reward). Here, rewards with a high positive reward component $r^p_t = +1$ have a probability of zero, that is, the only positive rewards that may be observed are of category -1 (low number of coins). Together, the rewards with $r^p_t = -1$ have a probability of one, that is, the observation of a low number of coins occurs on every trial for this reward state. Finally, the reward with $r^p_t = -1$ and $r^n_t = -1$ has a probability of 0.8 and the reward with $r^p_t = -1$ and $r^n_t =+1$ has a probability of 0.2. This means that the presence of electrotactile stimulation is of probability as defined in the “Experimental Design” section.

In summary, the task model describes the task as a trial-by-trial evolution of reward states $s_1,...,s_T$ taking on four possible values that encode semi-deterministic trial-by-trial associations between visual search field patterns $o_1,...,o_T$ and rewards $r_1,...,r_T$ based on $p^{s_t}(o_t)$ and $p^{s_t}(r_t)$. Note that from the agent’s perspective, the task reward states $s_t$ are latent, that is, only indirectly observable, while the visual search field patterns $o_t$ and the rewards $r_t$ are directly observable. The agent’s task is to decide on the trial-specific reward state. In the context of a behavioral model, this decision is then made observable as a trial-specific contingency rating (i.e., action). For implementational details of the task model, please refer to the Python script *abm_task.py*. Note that in line with Python’s offset-based indexing scheme, at the implementational level, trial indices run from 0 to 39, while for documentation purposes, we prefer the natural way of counting from 1 onward, such that t=1 refers to the first trial.

**Table 1:** Agent model space

	Agent type	Learning model parameters	Decision model dependencies
A0	Control	−	−
A1	Heuristic	−	Observed positive reward
A2	Heuristic	−	Observed positive and negative reward
A3	Rescorla-Wagner	Learning rate η	Expected positive and negative reward
A4	Rescorla-Wagner	Positive reward learning rate $\eta _p$, negative reward learning rate $\eta _n$	Expected positive and negative reward
A5	Rescorla-Wagner	Learning rate η, prior expectation bias π	Expected positive and negative reward
A6	Rescorla-Wagner	−	Expected positive and negative reward
A7	Rescorla-Wagner	Prior expectation bias π	Expected positive and negative reward
A8	Hybrid	Negative reward learning rate $\eta _n$	Observed positive and expected negative reward

*Notes*. The table displays the agent model denominations (first column), the agent model types (second column), the free parameters of the agents’ learning models (third column), and the agents’ decision model dependencies (fourth column). Note that agent models A6 and A7 implement a trial-adaptive learning rate instead of a free learning rate parameter η

#### Agent Models

To model the participants’ trial-by-trial decisions for a given reward state, we designed nine agent models that we denote by A0 to A8 (see Table 1). In the following, we introduce these agents in terms of their general structure, their learning models (if applicable), and their respective decision strategies. For implementational details, please refer to the Python scripts *abm_agent.py* and *abm_definition.py*.

***Control Agent A0***

Control agent A0 models a uniform random choice strategy to decide on the latent reward states. Formally, agent A0 can be conceived as the tuple

4 $$\begin{aligned} \mathcal {A} := (D,\phi , p(\delta _t)), \end{aligned}$$

where $D:=\{0,1,2,3\}$ denotes the set of agent decisions *d* that correspond to the latent reward states with the same numerical encoding as for the elements of *S*, ϕ denotes the agent’s decision value function, and $p(\delta _t)$ denotes the agent’s decision distribution. Specifically, we define

5 $$\begin{aligned} \phi : D \rightarrow \mathbb {R}, d \mapsto \phi (d) := \frac{1}{|D|} =: v(d), \end{aligned}$$

thus assigning equal decision values of 1/4 to each of the four decision options. Note that here and in the following, we denote the values of ϕ by *v*(*d*) to ensure consistency with the implementation (cf. *abm_agent.py*). Agent A0’s decision distribution $p(\delta _t)$ is defined as

6 $$\begin{aligned} p(\delta _t = d) := v(d), \end{aligned}$$

thus resulting in a uniform random choice policy. Clearly, we could also have defined $p(\delta _t)$ without using the agent’s decision value function, but we do so to emphasize the agent’s conceptual similarity to the remaining agent models, which encode deterministic decision strategies.

***Heuristic Agents A1 and A2***

Both heuristic agents A1 and A2 decide on the latent reward state solely based on the reward information available to them on a given trial. Specifically, agent A1 models the reward state decisions according to the observed level of positive reward and a post-decision random selection between the remaining two action options, which are distinguished by their respective levels of negative reward. Agent A2, on the other hand, decides on the latent reward state according to the observed level of both the positive and negative reward components. For example, if a low number of coins and the presence of electrotactile stimulation are observed on a given trial, agent A1 will decide on the latent reward state as d=0 (low positive reward, low negative reward) or d=2 (low positive reward, high negative reward) and let the post-decision noise make the final commitment to an action (cf. “BehavioralModels” section), while agent A2 will decide on d=2 (low positive reward, high negative reward). Formally, the agent model structure of agents A1 and A2 corresponds to the tuple

7 $$\begin{aligned} \mathcal {A} := ((D,\phi ,\delta ), r_t), \end{aligned}$$

where $(D,\phi ,\delta )$ denotes the agent’s decision model and $r_t$ denotes the fact that the agents have direct access to the trial-by-trial observed positive and negative reward components. In the agent’s decision model, *D* denotes the set of the agent’s decisions *d* as for agent A0, ϕ denotes the agent’s decision value function, and δ denotes the agent’s decision function. Note that these agents do not use any learning model or represent visual search field pattern observations $o_t$, which means that they are not able to store information on the association of visual search field patterns and reward states across trials.

For agent A1, the decision value function takes the form

8 $$\begin{aligned} \phi \!:\! D \!\rightarrow \! \mathbb {R}, d \mapsto \phi (d) \!:=\! {\left\{ \begin{array}{ll} 2 & (d = 0 \wedge r^p_t = -1) \\ 2 & (d = 1 \wedge r^p_t = +1) \\ 2 & (d = 2 \wedge r^p_t = -1) \\ 2 & (d = 3 \wedge r^p_t = +1) \\ 0 & \text {otherwise} \end{array}\right. } =: v(d), \end{aligned}$$

assigning higher decision values to those decisions that are in line with the trial-specific observed level of positive reward. According to (8), if the observed positive reward component $$r^p_t$$ is equal to -1, both ϕ(0)=2 and ϕ(2)=2. Similarly, if the observed positive reward $$r^p_t$$ is equal to +1, both ϕ(1)=2 and ϕ(3)=2. Note that setting ϕ(d) to a value of 2 for the agent’s most valued decision *d* and $\phi (d^{\prime })=0$ for the remaining decisions $d^{\prime }\neq d$ is arbitrary, as in the context of the behavioral model (cf. “Behavioral Models” section) any positive non-zero real number would result in the same action probability. Here and in similar cases below, we set the most valued decision to a value of 2 (instead of the perhaps more obvious choice of 1) to conceptually disentangle the decision value function values from the action probabilities, which are upper bounded by 1. Agent A1’s decision function is defined by

9 $$\begin{aligned} \delta : \Phi \rightarrow D^2, \phi \mapsto \delta (\phi ) := \text{ argmax}_{d \in D} \phi (d), \end{aligned}$$

where Φ denotes the decision value function space. As two values of ϕ are always identical (cf. (8)), for agent A1, $\text{ argmax}_{d \in D}$ always corresponds to a set of two elements of *D*.

For agent A2, the decision value function takes the form

10 $$\begin{aligned} \phi : D \rightarrow \mathbb {R}, d \mapsto \phi (d) :=\! {\left\{ \begin{array}{ll} 2 & (d = 0 \wedge r^p_t = -1 \wedge r^n_t = +1) \\ 2 & (d = 1 \wedge r^p_t = +1 \wedge r^n_t = +1) \\ 2 & (d = 2 \wedge r^p_t = -1 \wedge r^n_t = -1) \\ 2 & (d = 3 \wedge r^p_t = +1 \wedge r^n_t = -1) \\ 0 & \text {otherwise} \end{array}\right. } =: v(d), \end{aligned}$$

assigning higher decision values to those decisions that are in line with the trial-specific observed levels of positive and negative reward. The agent’s decision function is defined by

11 $$\begin{aligned} \delta : \Phi \rightarrow D, \phi \mapsto \delta (\phi ) := \text{ argmax}_{d \in D} \phi (d). \end{aligned}$$

Thus, agent A2 always decides on the latent reward state that is in line with the current trial’s observed rewards, which may or may not correspond to the actual task reward state given the probabilistic nature of the negative reward component.

***Rescorla-Wagner Learning-Based Agents A3 to A7***

Agents A3 to A7 implement a Rescorla-Wagner learning rule-based acquisition of the visual search field patterns’ associated expected reward values (Rescorla and Wagner, 1972; Sutton and Barto, 2018) and decide on that latent reward state in the direction of which these expected reward values have been biased. In contrast to the control agent A0 and the heuristic agents A1 and A2, agents A3 to A7 are therefore capable of incorporating information across trials by sequentially updating their knowledge based on newly acquired information. The agents’ general model structure corresponds to the tuple

12 $$\begin{aligned} \mathcal {A} := ((M, \psi ), (D, \phi , \delta ), (o_t,r_t)), \end{aligned}$$

where (M,ψ) denotes the agent’s learning model, $(D, \phi , \delta )$ denotes the agent’s decision model, and $(o_t,r_t)$ denotes the fact that the agents have direct access to the trial-by-trial visual search field pattern observations and observed rewards. Intuitively, the learning model (M,ψ) encodes trial-by-trial expected reward value estimates for each visual search field pattern and their updates based on the Rescorla-Wagner rules’ prediction error-correcting learning mechanism. The decision model $(D, \phi , \delta )$ implements a trial-by-trial decision for the reward state that is most plausible given the agent’s currently available information. The decision models of agents A3 to A7 are identical, while their learning models differ. In the following, we first unpack the agent-specific learning models and then make their common decision model explicit.

*Learning models* The learning model (M,ψ) of each agent comprises an expected reward value estimate space $M:= \mathbb {R}^8$ and a parameter-dependent expected reward value estimate update function ψ. The elements $\hat{\mu }$ of *M* are the vectors of the scalar visual search field pattern- and reward component-dependent expected reward value estimates, defined as

13 $$\begin{aligned} \hat{\mu } := \begin{pmatrix} \hat{\mu }^{o = 0}_{r^p}\\ \hat{\mu }^{o = 0}_{r^n}\\ \hat{\mu }^{o = 1}_{r^p}\\ \hat{\mu }^{o = 1}_{r^n}\\ \hat{\mu }^{o = 2}_{r^p}\\ \hat{\mu }^{o = 2}_{r^n}\\ \hat{\mu }^{o = 3}_{r^p}\\ \hat{\mu }^{o = 3}_{r^n}\\ \end{pmatrix} . \end{aligned}$$

Here, $\hat{\mu }^{o}_{r^p}$ and $\hat{\mu }^{o}_{r^n}$ refer to the expected positive and expected negative reward value estimates for a specific visual search field pattern *o*, respectively. For example, the third entry $\hat{\mu }^{o = 1}_{r^p}$ of $\hat{\mu }$ represents the agent’s expected positive reward value estimate for the visual search field pattern o=1.

ψ denotes the parameter-dependent expected reward value estimate update function, which transforms an agent’s expected reward value estimate on trial t-1 to the agent’s expected reward value estimate on trial *t* based on the observed visual search field pattern $o_t$ and the observed reward $r_t$. Formally, ψ is thus given by

14 $$\begin{aligned} \psi \!:\! O \times R \times M \!\rightarrow \! M, (o_t, r_t, \hat{\mu }_{t-1}) \!\mapsto \! \psi (o_t, r_t, \hat{\mu }_{t-1})\! =: \hat{\mu }_t. \end{aligned}$$

For t=1,...,T, the value of ψ is defined according to the Rescorla-Wagner learning rule as

15 $$\begin{aligned} \psi (o_t, r_t, \hat{\mu }_{t-1}) := \hat{\mu }_{t-1} + \eta \cdot (\tilde{r}_t - \tilde{\mu }_{t-1}), \end{aligned}$$

where

16 $$\begin{aligned} \tilde{r}_t := e_{o_t+1} \otimes r_t, \quad \tilde{\mu }_{t-1} := \left( e_{o_t+1} \otimes 1_2 \right) \circ \hat{\mu }_{t-1}. \end{aligned}$$

Here, η>0 denotes the learning rate parameter that scales the agent’s trial-dependent reward prediction error $(\tilde{r}_t - \tilde{\mu }_{t-1})$. The Kronecker and Hadamard products as well as the use of the 4-dimensional canonical unit vector $e_{o_{t+1}}$ in (16) serve to select the appropriate positive and negative expected reward value estimates for the observed visual search field pattern on trial *t* as detailed in Appendix C. Importantly, the definitions of both the initial expected reward value estimate $\hat{\mu }_0$ and the learning rate η differ between the five Rescorla-Wagner learning-based agents.

*Initial expected reward value estimates* Agents A3, A4, and A6 assign an equal initial expected reward value of 0 to each visual search field pattern and reward component such that for these agents, $\hat{\mu }_0:= 0_8$.

In contrast, agents A5 and A7 encode a parameterized initial expected negative reward bias. Specifically, for agents A5 and A7

17 $$\begin{aligned} \hat{\mu }_0 := \begin{pmatrix} 0 \\ \pi \\ 0 \\ \pi \\ 0 \\ \pi \\ 0 \\ \pi \end{pmatrix}, \end{aligned}$$

where $\pi \in [-1,1]$ denotes a free prior expectation bias parameter. The inclusion of π is motivated as a means for capturing the experimentally observed participants’ inductive bias to decide for low negative reward states when in fact high negative reward states are presented, in particular during early trials of the experiment (cf. “Descriptive Analyses” section). In conjunction with the agents’ decision value function (cf. (21)), positive values of π bias the agents’ decision towards low negative reward state decisions, even if high negative reward values are observed by the agent. For a numerical example with π=0.2, see Appendix E (E.3 Agent A5). Note that in its data-analytical application, π is treated as a free parameter and thus allows for capturing sub-rational expectations, but does not endow the agent models with a different form of prior knowledge than the participants.

*Learning rate parameters* In addition, the Rescorla-Wagner learning-based agents differ in terms of their learning rate parameter η. While η is a scalar positive real number for agents A3 and A5, for agent A4, it is given by

18 $$\begin{aligned} \eta := e_{o_t+1} \otimes \begin{pmatrix} \eta _p \\ \eta _n \end{pmatrix}, \end{aligned}$$

where $\eta _p> 0, \eta _n > 0$ denote positive and negative reward component-specific learning rate parameters that allow for differential updates of the expected positive and negative reward value estimates. The canonical unit vector Kronecker product in (18) again serves the selection of the visual search field pattern-specific expected reward value estimates. Finally, for agents A6 and A7, the size of the expected reward value estimate updates depends on the number of trials already completed and, for t=1,...,T, is defined as

19 $$\begin{aligned} \eta := \frac{1}{t}, \end{aligned}$$

such that the scaling of the reward prediction error decreases over trials from η=1 on trial t=1 to η=0.025 on trial t=40. In doing so, more importance is assigned to initial observations when the agent’s uncertainty about the visual search field patterns’ associated reward states is maximal, and it is less pronounced when information about said associated reward states has already been accrued by the agent. Note that the learning rates of agents A3, A4, and A5 are free model parameters, while the definition of the trial-adaptive learning rate (19) implies that the learning rate of agents A6 and A7 is not a free parameter.

*Decision models* For all Rescorla-Wagner learning-based agents A3 to A7, the agent’s decision model is given by

20 $$\begin{aligned} (D, \phi , \delta ). \end{aligned}$$

Here, *D* and δ are identical to the definitions for agents A1 and A2, but the agents’ decision value function ϕ differs. In general, agents A3 to A7 base their decisions on their respective expected reward value estimates and assign higher decision values to those decisions for which the direction signs of the expected reward value estimates are in line with the latent task reward states. Formally, the decision value function of agents A3 to A7 is defined as

21 $$\begin{aligned} \phi : D \rightarrow \mathbb {R}, d \mapsto \phi (d) := {\left\{ \begin{array}{ll} 2 & (d = 0 \wedge \hat{\mu }^o_{r^p}< 0 \wedge \hat{\mu }^o_{r^n} \ge 0) \\ 2 & (d = 1 \wedge \hat{\mu }^o_{r^p} \ge 0 \wedge \hat{\mu }^o_{r^n} \ge 0) \\ 2 & (d = 2 \wedge \hat{\mu }^o_{r^p}< 0 \wedge \hat{\mu }^o_{r^n}< 0) \\ 2 & (d = 3 \wedge \hat{\mu }^o_{r^p} \ge 0 \wedge \hat{\mu }^o_{r^n} < 0) \\ 0 & \text {otherwise} \end{array}\right. } =: v(d). \end{aligned}$$

Together with the decision function δ, (21) encodes the following decision-making mechanism. On a given trial, the values of the decision value function $\phi (d), d \in D$ depend on the two expected reward value estimates $\hat{\mu }^o_{r^p}$ and $\hat{\mu }^o_{r^n}$ of the observed visual search field pattern *o*. The agent then allocates a value of ϕ(d)=2 to a specific decision *d*, if these expected reward value estimates have previously been biased in the direction of a given visual search field pattern’s associated reward. Specifically, if, for example, $\hat{\mu }^o_{r^p} \ge 0$ and $\hat{\mu }^o_{r^n} \ge 0$, the expected reward value estimates for visual search field pattern *o* have been biased in the direction of the reward with $r^p = +1$ and $r^n = +1$. The reward $r = (+1,+1)^T$ in turn corresponds to the reward most indicative of reward state $s_t = 1$ (high positive reward, low negative reward) and, upon evaluation of the decision function δ, the agent will decide on $d_t = 1$. Because the conditions on $\hat{\mu }^o_{r^p}$ and $\hat{\mu }^o_{r^n}$ are mutually exclusive, the agent will always favor one and only one decision based on the expected reward value estimates $\hat{\mu }^o_{r^p}$ and $\hat{\mu }^o_{r^n}$ for the observed visual search field pattern *o*.

***Hybrid Agent A8***

Finally, agent A8 combines a Rescorla-Wagner learning model with a heuristic decision strategy. Specifically, for this agent, the expected negative reward component values are learned, whereas the positive reward component is not learned but chosen based on the trial-specific reward observation. That is, if a low number of coins is observed, agent A8 will assign higher decision values to low positive reward states, and if a high number of coins is observed, the agent will assign higher decision values to high positive reward states. As for agents A3 to A7, this agent’s general model structure corresponds to the tuple

22 $$\begin{aligned} \mathcal {A} := ((M, \psi ), (D, \phi , \delta ), (o_t,r_t)), \end{aligned}$$

where (M,ψ) denotes the agent’s learning model, $(D, \phi , \delta )$ denotes the agent’s decision model, and $(o_t,r_t)$ indicates the agent’s direct access to the trial-specific visual search field pattern observations and observed positive and negative reward components. The learning model (M,ψ) of agent A8 is similar, but not identical, to that of the Rescorla-Wagner learning-based agents A3 to A7. Specifically, agent A8’s free learning rate parameter is given by

23 $$\begin{aligned} \eta := e_{o_t+1} \otimes \begin{pmatrix} 0 \\ \eta _n \end{pmatrix}, \end{aligned}$$

where $\eta _n > 0$ and in contrast to agent A4 (cf. (18)), the positive reward learning rate parameter is fixed to $\eta _p:= 0$. Regarding the agent’s A8 decision model $(D,\phi ,\delta )$, the decision set *D* and the decision function δ are identical to those of agents A3 to A7, while the agent’s hybrid heuristic/learning-based decision value function is defined as

24 $$\begin{aligned} \phi : D \rightarrow \mathbb {R}, d \mapsto \phi (d) :=\! {\left\{ \begin{array}{ll} 2 & (d = 0 \wedge r^p = -1 \wedge \hat{\mu }^o_{r^n} \ge 0) \\ 2 & (d = 1 \wedge r^p = +1 \wedge \hat{\mu }^o_{r^n} \ge 0) \\ 2 & (d = 2 \wedge r^p = -1 \wedge \hat{\mu }^o_{r^n}< 0) \\ 2 & (d = 3 \wedge r^p = +1 \wedge \hat{\mu }^o_{r^n} < 0) \\ 0 & \text{ otherwise } \end{array}\right. } \!=: v(d). \end{aligned}$$

Crucially, the decision values of agent A8 thus depend on both the observed positive reward component $r^p$ on a given trial as well as the accrued information on the expected negative reward value estimate.

#### Behavioral Models

Agent models A1 to A8 are deterministic theories on how participants might learn the associations between visual search field patterns and reward states and decide accordingly. In particular, given a fixed sequence of reward states, visual search field pattern observations, and rewards, these agents predict a fixed sequence of reward state decisions. In light of experimental data, a single empirically observed deviation from an agent’s model reward state decision would thus invalidate the theory embodied by a given agent. Thus, to encode our uncertainty about the proposed theories and to allow for residual variance beyond the models’ deterministic predictions, we embed the agent models’ decision value functions in parameterized action probability distributions and refer to the joint compositions of task model, agent models, and their probabilistic embedding as *behavioral models*. Like any models used for explaining empirical data, the thus constructed models can be considered in a data-generative and data-analytical setting. In a data-generative setting, for fixed model parameters, the models allow for sampling actions whose descriptive statistics can be analyzed and compared with experimentally observed data to assess the models’ face validity. In a data-analytical setting, for fixed (simulated or experimentally observed) data, the model parameters can be estimated and the models subjected to model comparison procedures. Formally, for agent models *A*1 to *A*8, the behavioral models take the form

25 $$\begin{aligned} \mathcal {B} := (\mathcal {T}, \mathcal {A}, A, p^{\tau }(a_t)), \end{aligned}$$

where

- $\mathcal {T}$ denotes the task model,
- $\mathcal {\mathcal {A}}$ denotes the agent model *A*1 to *A*8 of interest,
- $A:= \{0,1,2,3\}$ denotes the set of observable contingency rating actions *a* with the same numerical encoding as the task reward states and the agent’s decisions,
- $p^{\tau }(a_t)$ denotes the action probability distribution, which is based on the agent’s decision value function and is dependent on a post-decision noise parameter. It is formally defined as
  26 $$\begin{aligned} p^{\tau }(a_t = a) := \frac{\text{ exp }(\tau ^{-1}v(a))}{\sum _{a \in A} \text{ exp }(\tau ^{-1}v(a))}, \end{aligned}$$
  with v(a):=v(d) for a=d and where τ>0 denotes the post-decision noise parameter. Note that the functional form of the right-hand side of (26) is commonly referred to as *softmax function* or *normalized exponential* (Bishop, 2006). It transforms a set of discrete function values into a set of probability values that sum to one in a parameter-dependent manner. For large values of the post-decision noise parameter τ, the resulting probability mass function over actions *a* approaches a discrete uniform distribution, while for very small values of τ, the resulting probability values are proportional to the values assigned to the actions by the decision value function with one value dominating. In other words, with τ approaching values near 0, the agents’ actions will most frequently equal the decision option with the highest decision value. Intuitively, sampling from the resulting probability distribution can therefore be considered a noisy argmax operation and an approximation of the agents’ decision function δ (cf. Reverdy and Leonard, 2016).

Finally, as discussed above, the agent model A0 is a stochastic theory about the participants’ decision strategy. The empirically observed actions of this model can thus be directly evaluated under this model by setting

27 $$\begin{aligned} p(a_t = a) := v(a) \text{ for } a = d. \end{aligned}$$

Notably, a parameter-dependent embedding of this agent’s decision value function akin to (26) would result in a uniform probability distribution identical to the control agent’s decision distributions for any value of the parameter τ, rendering the respective parameter non-identifiable. We elaborate on this in Appendix F. Intuitively, given a set of observed contingency rating actions, it is not possible to distinguish whether these have been generated by a decision strategy favoring a single decision, but imbued with a high level of post-decision noise, rendering actions distributed uniformly at random, or by a uniform random decision strategy. Note that for implementational convenience, the behavioral models described in this section are implemented within the Python script *abm_agent.py* using the function definitions *p_a* and *rvs_a*.

In summary, the formulation of the behavioral models according to (25) and their Python implementation furnish a generative framework that can emulate the experimentally acquired participant behavior under different assumptions about the participants’ learning and decision-making strategies (which are cast as agent models). In the following sections, we consider this framework from both a data-generative and data-analytical perspective. To this end, we denote the union of sequences of reward states, visual search field patterns, rewards, and actions for a given participant *i* with i=1,...,n by $\mathcal {Y}_i$, and refer to $\mathcal {Y}_i$ as a *data set*.

### Parameter Estimation and Model Comparison

To determine which behavioral model is most likely to have generated an experimental or simulated data set, it is necessary to first evaluate the best-fitting set of parameter values for each behavioral model and then compare the models with their best-fitting parameter values in terms of their data fit and complexity. In the following, we will outline the general parameter estimation and model comparison procedure applied in the present work. Note that throughout the “ModelValidation” section, the estimation and comparison procedures were performed based on simulated data, that is, data that were artificially generated using a specific known behavioral model with a particular known setting of parameter values. In the “Evaluation of the Experimental Data” section, the behavioral models were then evaluated on the basis of the actual experimental data acquired from the participants.

To estimate the behavioral model-specific sets of free parameters θ, we applied a maximum likelihood approach. Specifically, for each (experimental or simulated) data set, we evaluated the negative log likelihood function by summing the negative log probabilities of the trialwise actions under a given behavioral model for a specific value of θ. In particular, the action probabilities were retrieved from the action probability distribution $p^\tau (a_t)$ as defined in (26). Note that depending on the behavioral model considered, the log likelihood function is a function of none to three scalar parameters. Specifically, as the control agent A0 implements a fully uniform random choice policy, no parameters must be estimated for this behavioral model as explained in the “Behavioral Models” section and the log likelihood can be evaluated directly using (27). For the behavioral models of agents A1, A2, and A6, the log likelihood function is a function of the post-decision noise parameter $\tau \in [0.01, 2]$ only. Thus, for these behavioral models $\theta := \tau$. For the behavioral models of agents A3 and A8, the log likelihood function is a function of the learning rate parameter $\eta \in [0.01, 2]$ and the post-decision noise parameter $\tau \in [0.01, 2]$, rendering $\theta := (\eta , \tau )$ a two-dimensional vector. For the behavioral model of agent A4, the log likelihood function is a function of the positive and negative reward learning rate parameters $\eta _p, \eta _n \in [0.01, 2]$ and the post-decision noise parameter $\tau \in [0.01, 2]$. Thus, for this behavioral model $\theta := (\eta _p, \eta _n, \tau )$ is a three-dimensional vector. For agent A5, the log likelihood function is a function of the learning rate parameter $\eta \in [0.01, 2]$, the prior expectation bias parameter $\pi \in [-1, 1]$, and the post-decision noise parameter $\tau \in [0.01, 2]$, rendering $\theta := (\eta , \pi , \tau )$ a three-dimensional vector. For agent A7, the log likelihood function is a function of the prior expectation bias parameter $\pi \in [-1, 1]$ and the post-decision noise parameter $\tau \in [0.01, 2]$. Thus, for this behavioral model $\theta := (\pi , \tau )$ is again a two-dimensional vector.

For every behavioral model, we estimated θ based on a single data set $\mathcal {Y}_i$ by minimizing the respective negative log likelihood function using SciPy’s minimization function *minimize* with the Nelder-Mead algorithm as optimization method (Gao and Han, 2012; Virtanen et al., 2020). The parameter constraints were set to $\tau , \eta , \eta _p, \eta _n \in [0.01, 2]$ and $\pi \in [-1, 1]$. To reduce the risk of identifying local minimizers rather than global minimizers of the negative log likelihood function, the initial parameter estimate values were selected as evenly spaced within the respective parameter space boundaries, and the parameter estimation procedure was repeated for a predefined number of initial parameter estimate values. Specifically, we intended to achieve an approximately equal number of initial parameter estimate values for all behavioral models, regardless of the dimension of their parameter spaces. For the analyses in the  “Model Validation” and “Post-Hoc Model Validation” sections, we selected $100^{\frac{1}{n_p}}$ evenly spaced initial parameter estimate values for each parameter of the respective behavioral model, where $n_p$ refers to the dimensionality of the behavioral model’s parameter space. This procedure resulted in 100 (for one- and two-dimensional parameter spaces) or 125 initial parameter estimate values (for three-dimensional parameter spaces). For the analyses in the “Evaluation ofthe Experimental Data” section, we selected $1000^{\frac{1}{n_p}}$ evenly spaced initial parameter estimate values for each parameter of the respective behavioral model, resulting in 1000 or 1250 initial parameter estimate values. Across all repetitions of the parameter estimation procedure, only the parameter estimate values achieving the smallest negative log likelihood function value were regarded as ML estimates (Wilson and Collins, 2019; Horvath et al., 2021). In the case of multiple local minimizers with equal negative log likelihood function values, the parameter estimate value with the smallest Euclidean norm was selected. If the parameter estimate values did not differ in terms of their Euclidean norm, the first identified minimizer was considered.

**Fig. 5:**
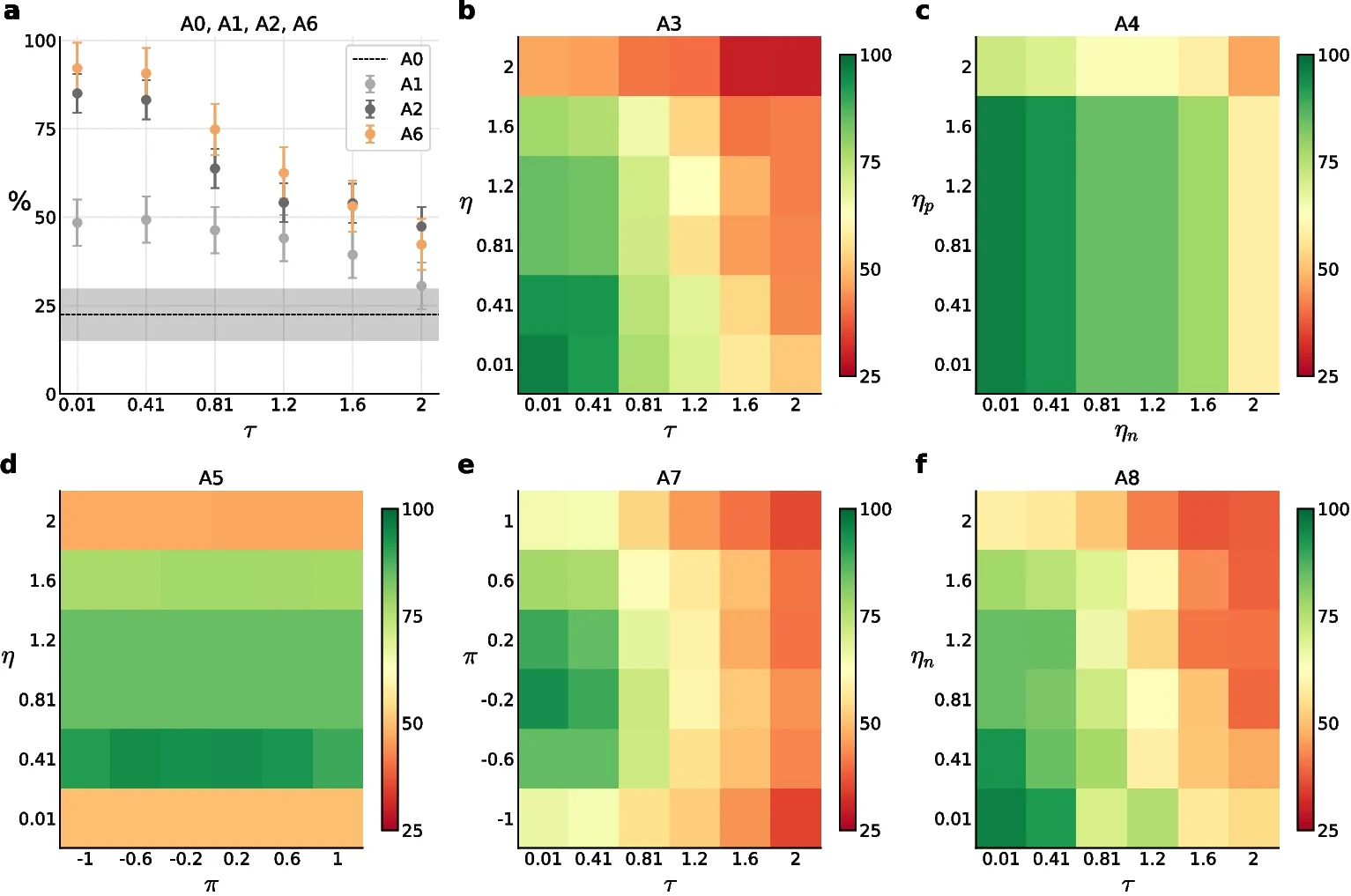
Simulated behavior of our behavioral models. **a** Average action accuracies observed for the behavioral models embedding agent A0 (dashed line), A1 (light gray), A2 (dark gray), and A6 (orange), respectively. The light gray area around the dashed line and whiskers correspond to standard deviations (*SD*s). **b** Average action accuracy observed for the behavioral model embedding the Rescorla-Wagner learning-based agent A3. **c** Average action accuracy observed for behavioral model embedding the Rescorla-Wagner learning-based agent A4 for a post-decision noise parameter of τ=0.01. **d** Average action accuracy observed for the behavioral model embedding the Rescorla-Wagner learning-based agent A5 for a post-decision noise parameter of τ=0.01. **e** Average action accuracy observed for the behavioral model embedding the Rescorla-Wagner learning-based agent A7. **f** Average action accuracy observed for the behavioral model embedding the hybrid agent A8. All parameter values were rounded to two decimal places. For the generation of this figure, please refer to *abm_figure_5.py*

To compare the behavioral models’ relative plausibilities given the experimental or simulated data, we first evaluated all model- and data set-specific Bayesian Information Criterion scores according to Schwarz (1978)

28 $$\begin{aligned} \text {BIC}_{ij} := \ell _{ij}(\hat{\theta }) - \frac{k_j}{2} \ln T, \end{aligned}$$

where i=1,...,n indexes data sets, $j = \text {A0,..., A8}$ indexes models, $\ell _{ij}$ denotes the log likelihood function of model *j* for data set *i*, $\hat{\theta }$ denotes the model- and data set-specific ML parameter estimate, $k_j$ denotes the number of free model parameters and T=40 denotes the total number of trials per data set. Note that according to (28), higher BIC values indicate higher model plausibilities. As a first group-level indicator for the most plausible model, we summed the model-specific BIC values across all data sets, corresponding to the approximated log joint probability of all data sets under the given model and the assumption of pairwise data set independence. We then subjected the BIC values of all agents and data sets to a random-effects Bayesian model selection procedure using a Python implementation of the protected exceedance probability (PEP) approach as proposed by Stephan et al. (2009) and Rigoux et al. (2014). For each model, the Python script *abm_bmc.py* returns a PEP, which corresponds to the group-level probability that this model is the most likely among all models considered given the data. Formally,

29 $$\begin{aligned} \text {PEP}_j:= p\left( \mathcal {B}_j = \max \{\mathcal {B}_{A0},...,\mathcal {B}_{A8} \}|\mathcal {Y}_1,...,\mathcal {Y}_n\right) \text { for } j = \text {A0, ..., A8}, \end{aligned}$$

where $\text{ PEP}_j$ denotes the PEP of the behavioral model $\mathcal {B}_{j}$ that embeds agent model $j = \text {A0,..., A8}$, and $\mathcal {Y}_1$,..., $\mathcal {Y}_n$ denote the (experimental or simulated) data sets. Note that in contrast to a BIC score summation approach, for a given behavioral model, the PEP approach accounts for the uncertainty that different data sets may have been generated by different models of the model space. For further implementational details on the parameter estimation and model comparison procedure, please refer to the Python scripts *abm_estimation.py* and *abm_log_likelihood.py*.

### Model Validation

To validate the agent-based behavioral modeling approach, we performed simulations that assessed the models’ face validity, recoverability, and parameter identifiability. Specifically, we first generated artificial data sets using each of the behavioral models in a generative setting. To this end, we employed the experimentally delivered reward state, visual search field pattern, and reward sequences of a given participant and sampled agent-specific actions on 40 trials. For the parameter-free agent A0, the simulated data were generated without further specifications. For the remaining behavioral models, we opted for a coverage of the parameter space that balanced computational efficiency and theoretical insight. We thus decided on a resolution of six evenly spaced parameter values for each component of θ. This resulted in six true, but unknown, parameter values for models with a one-dimensional parameter space (behavioral models of agents A1, A2, A6) and 36 true, but unknown, parameters for models with a two-dimensional parameter space (behavioral models of agents A3, A7, and A8). As for a three-dimensional parameter space the full parameter space coverage with 216 true, but unknown, parameters exceeded our computational model validation resources, we focused on model validation in a low post-decision noise scenario for the behavioral models of agents A4 and A5. For our generative simulations, we selected post-decision noise parameter values $\tau \in \{0.01, 0.408, 0.806, 1.204, 1.602, 2\}$, learning rate parameter values $\eta , \eta _p, \eta _n \in \{0.01, 0.408, 0.806, 1.204, 1.602, 2\}$, and prior expectation bias parameter values $\pi \in \{-1, -0.6,$ -0.2,0.2,0.6,1}, and performed the data generation procedure for all possible combinations, except for the behavioral models of agents A4 and A5, where we used a fixed post-decision noise parameter of τ:=0.01. For each data-generating model and each parameter value, we then generated 50 data sets, corresponding to the experimental sample size and using all reward state, visual search field pattern, and reward sequences as employed in the experiment. For implementational details, please refer to the Python scripts *abm_validation.py* and *abm_validation_run.py*.

#### Model Face Validity

To validate the formulation and implementation of our model set and to explore the models’ behavioral repertoires, we evaluated the average action accuracy of each behavioral model and parameter setting. We visualize the results in Fig. 5.

Figure 5a depicts the average action accuracies for the behavioral models with a zero- and one-dimensional parameter space (A0, A1, A2, A6). As discussed above, agent A0 implements a uniform random choice policy. Given the four action options, we thus expected an average action accuracy of approximately 25% for this model and indeed observed an average action accuracy of 22.4% across the simulated data sets. For the behavioral models of agents A1, A2, and A6, the average action accuracy decreased with increasing values of the post-decision noise parameter τ, slowly converging towards a uniform random choice policy. This simulation result is in line with (26), which implies that higher values of τ correspond to lower probabilities that the decision with the highest decision value is realized as an action. Because agent A1 implements a heuristic choice policy for the observed level of positive reward, we expected an average action accuracy of approximately 50% in the absence of post-decision noise for this model. In other words, if the agent’s action is identical to its decision, agent A1 should always perform correctly for the reward state-indicative positive reward. Consistent with this expectation, an average action accuracy of 48.4% was observed for agent A1 at the lowest post-decision noise level of τ=0.01. Since agent A2 implements a heuristic choice policy for the observed level of both positive and negative reward, we expected the agent to only emit incorrect actions in the presence of reward state-non-indicative observations. As described in the “Experimental Design” section, the observed negative reward was not always indicative of the reward state. Specifically, for low negative reward states, 1 of 10 repetitions included electrotactile stimulation, while for high negative reward states, 2 of 10 repetitions did not include electrotactile stimulation. In total, participants and agents were thus faced with six reward state-non-indicative observations. When following the heuristic choice policy of agent A2, we thus expected an average action accuracy of $34/40 \cdot 100 = 85\%$ in the absence of post-decision noise, which is in line with the observed average action accuracy of 85% at τ=0.01. For the Rescorla-Wagner learning-based agent A6, we observed a slightly better performance than for the heuristic agent A2 for small to medium values of τ. This minor performance difference can be explained by the nature of agent A6’s trial-adaptive learning rate parameter, which decreases from 1 to 0.025 across trials (cf. (19)). The smaller the learning rate, the less weight is assigned to the agent’s prediction error, i.e., the difference between its observed and estimated expected reward values. Consequently, if the agent encounters reward state-non-indicative observations in later trials, it will not necessarily make an incorrect decision, explaining the slightly higher average action accuracies when compared to agent A2.

Figure 5b depicts the average action accuracies for the behavioral model embedding the Rescorla-Wagner learning-based agent A3, which exhibits two parameter dependencies. First, as expected, the average action accuracy decreased with increasing values of the post-decision noise parameter τ. Second, the average action accuracy decreased with increasing values of the learning rate parameter η. As numerically illustrated in a trial-by-trial setting in Appendix E (E.1 Agent A3), this can be explained by the brittle nature of the learning rate parameter (cf. Zhang et al., 2020). Specifically, the higher the learning rate parameter value, the more the agent’s prediction error is weighted, which results in comparably large and highly varying trial-by-trial expected reward value estimate updates. As a result, with a learning rate parameter value of η=2, the agent’s decisions tend to vary from trial to trial independently of the observed rewards, largely reducing the average action accuracy. In contrast, for small learning rate parameter values, differences between observed and expected reward values are less heavily taken into account, resulting in a more stable reward state-specific decision behavior across trials.

Figure 5c depicts the average action accuracies of the behavioral model embedding the Rescorla-Wagner learning-based agent A4 as a function of its two reward component-specific learning rate parameters $\eta _p$ and $\eta _n$ for a fixed post-decision noise parameter value of τ=0.01. As expected and in line with the learning rate parameter-dependent behavior of agent A3, the higher the negative reward learning rate $\eta _n$, the lower the average action accuracy. Second, no accuracy differences were observed for values of the positive reward learning rate parameter $\eta _p$ between 0.01 and 1.602. However, the average action accuracy decreased from $\eta _p = 1.602$ to $\eta _p = 2$ for all values of $\eta _n$. The explanation of these observations is two-fold. First, the different effects of $\eta _p$ and $\eta _n$ on the average action accuracy arise from the experimental design. In contrast to the negative reward component, there is a one-to-one correspondence between the positive reward state and the observed positive reward. Thus, the correct identification of the reward state-indicative positive reward level does not require any learning processes beyond the first trial, explaining the relative independence of the average action accuracy from the value of the positive learning rate parameter $\eta _p$. However, as numerically illustrated in Appendix E (E.2 Agent A4), for the highest positive reward learning rate parameter value of $\eta _p = 2$, the induced oversensitivity towards differences between observed and expected reward values leads to instable, non-converging decision behavior, reducing the observed average action accuracy.

Figure 5d depicts the average action accuracies for the behavioral model embedding the Rescorla-Wagner learning-based agent A5 for a fixed post-decision noise parameter of τ=0.01. We first note that the action accuracy was highest for medium values of η and lowest for extreme values of η (i.e., η=0.01 and η=2). For medium to large values of η, we observed a decrease in action accuracy. Regarding the prior expectation bias parameter, similar effects on the action accuracy could be observed for both positive and negative values of π. This effect results from the fact that π decreases the action accuracy for high negative reward states, but leaves the accuracy for low negative reward states unaffected when it assumes a negative value, and vice versa when it assumes a positive value. We illustrate this effect numerically by trial-by-trial examples in Appendix E (E.3 Agent A5). Finally, for a small learning rate parameter of η=0.01, the initial expectation bias could not be compensated by subsequent reward observations, resulting in an overall low action accuracy.

Figure 5e depicts the average action accuracies for the behavioral model embedding the Rescorla-Wagner learning-based agent A7. Here, the average action accuracy decreased with increasing values of both the absolute prior expectation bias parameter π and the post-decision noise parameter τ. As described for agents A3 and A5, both effects are expected based on the formulation of the behavioral model of agent A7. Finally, Fig. 5f depicts the average action accuracies for the behavioral model of the hybrid agent A8. Here, increasing values of both the negative reward learning rate parameter $\eta _n$ and the post-decision noise parameter τ resulted in lower average action accuracies. Again, these effects are expected as explained for the previous behavioral models with similar parameter dependencies.

**Fig. 6:**
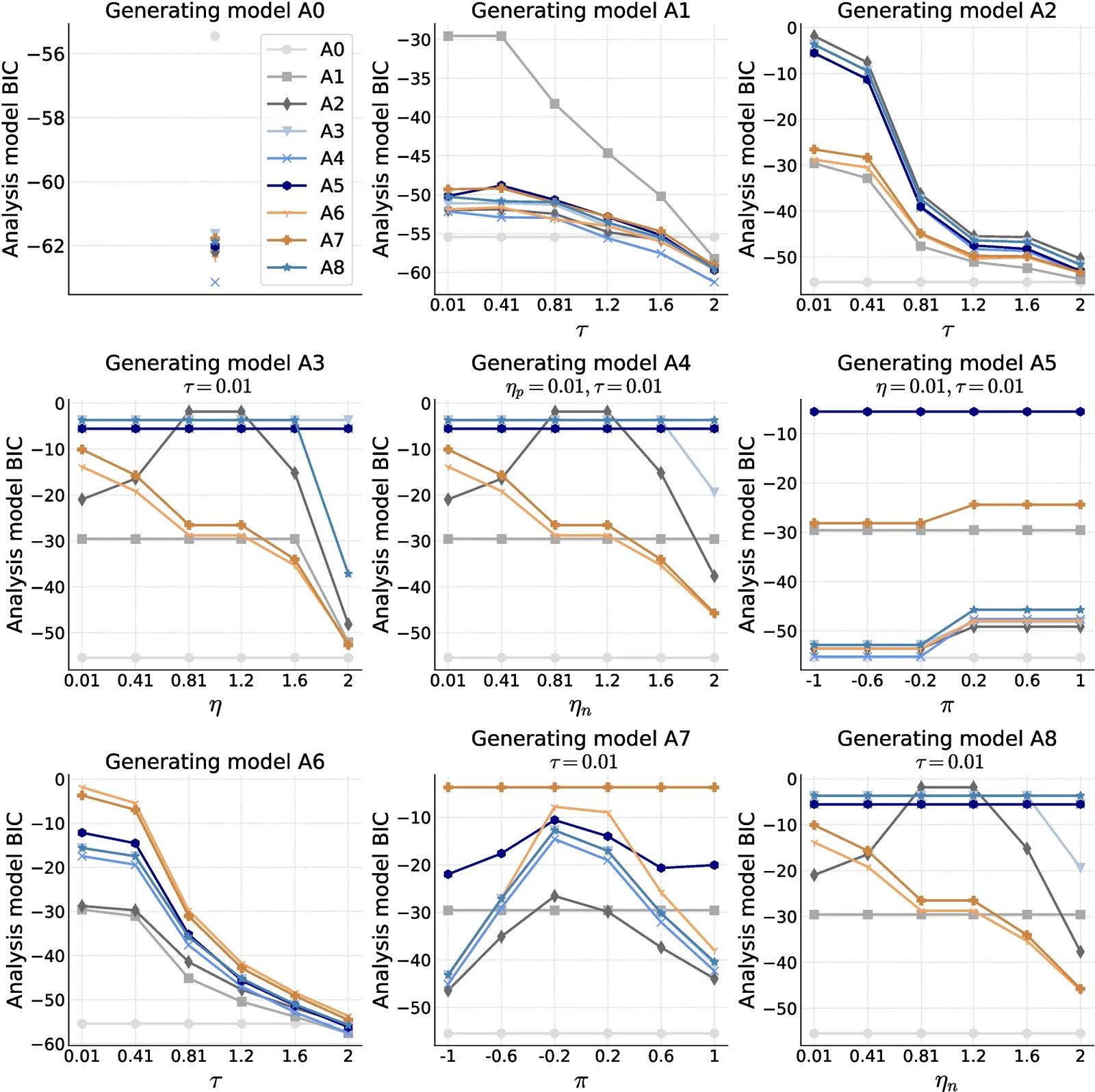
Model recovery analyses based on BIC values. Each panel refers to a data-generating model of our behavioral model space. For each data-generating process, all behavioral models served as data analysis model to allow for model comparison. Higher BIC values indicate higher model plausibility (cf. Schwarz, 1978). Behavioral models are differentiated in terms of colors and marker types as depicted in the leftmost upper panel. For multidimensional parameter spaces, at least one parameter was constrained to a fixed value to reduce complexity of results. All parameter values were rounded to two decimal places. For the generation of this figure, please refer to *abm_figure_6.py*

**Fig. 7:**
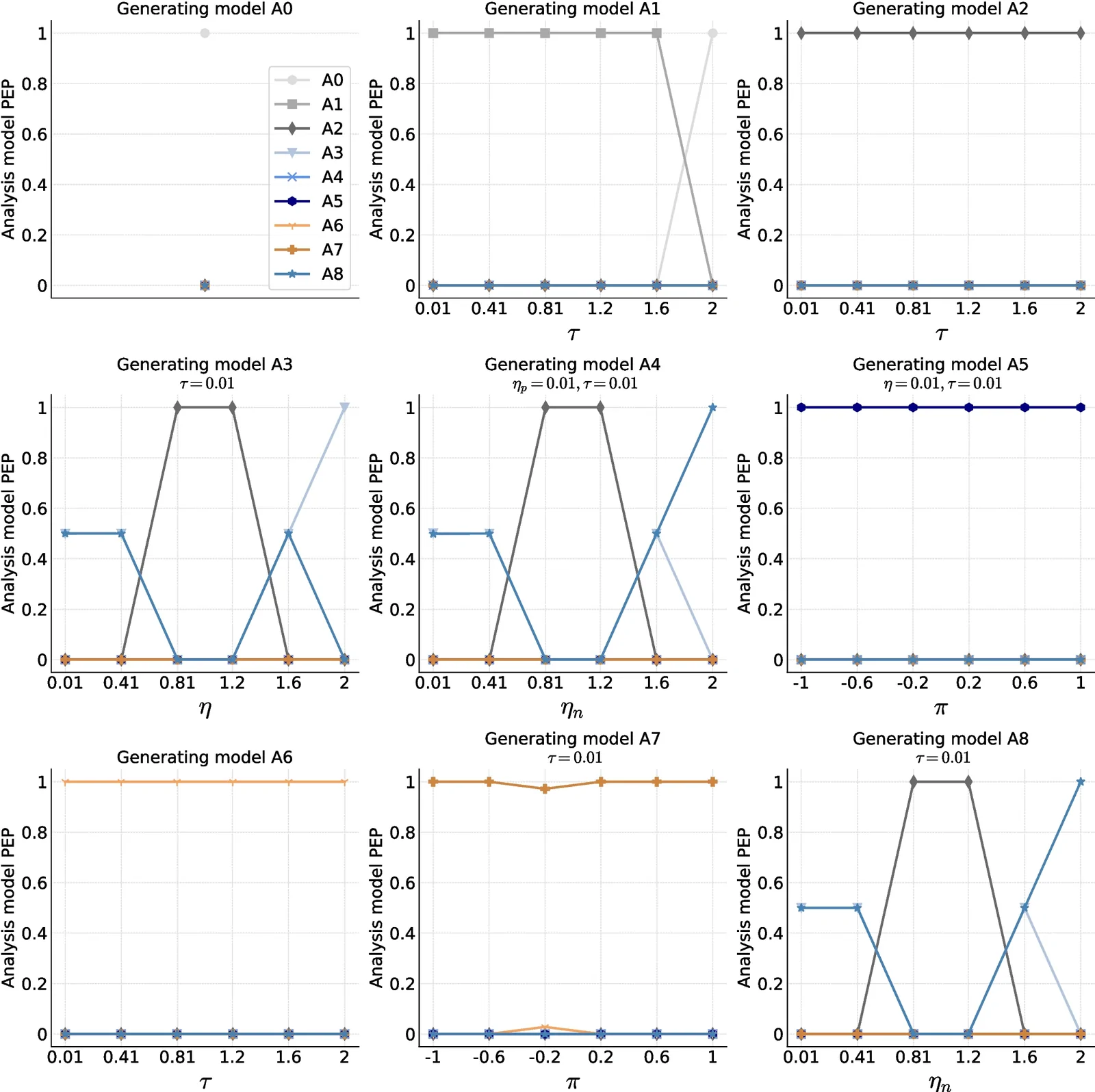
Model recovery analyses based on PEPs. Each panel refers to a data-generating model of our behavioral model space. For each data-generating process, all behavioral models served as data analysis model to allow for model comparison. Higher PEPs indicate higher model plausibility. Behavioral models are differentiated in terms of colors and marker types as depicted in the leftmost upper panel. For multidimensional parameter spaces, at least one parameter was constrained to a fixed value to reduce complexity of results. All parameter values were rounded to two decimal places. For the generation of this figure, please refer to *abm_figure_7.py*

#### Model Recovery

To assess the degree to which our behavioral models can be reliably distinguished, we performed a series of model recovery analyses based on the artificially generated data sets described above. Specifically, for each model in our model space, we analyzed the data generated by this model using all behavioral models by means of the procedures described in the  “Parameter Estimation and Model Com-parison” section. Figures 6 and 7 summarize the results of these analyses in terms of BIC values and PEPs, respectively. For each data-generating model, the corresponding panel depicts the BIC values or PEPs of each data analysis model. For the behavioral models based on agents A1 to A8, BIC values and PEPs are displayed as functions of one of the data-generating model’s parameters with the remaining data-generating model’s parameters set to constant values to reduce the complexity of the results presented. We justify the selection of the respective data-generating model’s parameter in the discussion below.

For data generated based on agent A0, the highest BIC value and highest PEP were achieved by the data analysis model based on agent A0, indicating that A0 can be reliably recovered. For data generated based on agents A1 and A2, the BIC values and PEPs were highest for the data analysis model based on agent A1 and agent A2, respectively, for all values of the generative post-decision noise parameter τ, except for the highest level of τ. Specifically, for τ=2, the highest BIC value and highest PEP for data generated based on agent A1 were achieved by the data analysis model based on agent A0. Notably, data generated based on agent A1 with a high level of post-decision noise resembles the uniform random choice policy of A0, which can explain such data with one parameter fewer than A1. Overall, these results indicate that the behavioral models based on agents A1 and A2 can be reliably recovered.

For data generated based on agent A3, Figs. 6 and 7 visualize the model recovery analyses results across all values of the learning rate parameter η for a post-decision noise parameter of τ=0.01, as larger post-decision noise naturally leads to a reduction in model identifiability (cf. model recovery analyses agents A1 and A2). For η<0.806 and η=1.602, the highest BIC values and highest PEPs were achieved by the data analysis models based on both agent A3 and agent A8. Notably, these models differ only in terms of the definition of the learning rate parameter. While agent A8’s learning parameter is restricted to the negative reward component, agent A3’s learning rate parameter concerns both the positive and negative reward components. However, due to the deterministic nature of the positive reward distribution, acquiring the association of visual search field patterns and the positive reward state does not require any learning processes beyond their first joint encounter. Agent A8 can thus mimic the behavior of model A3, and both agent models exhibited identical model plausibility values when the data were generated based on agent A3. For medium values of the learning rate parameter, the behavioral model based on agent A2 showed the highest BIC values and highest PEPs. Notably, for these learning rate parameter values, agent A2 generates behavior similar to that of agent A3. As explained in the “Model Face Validity” section, for medium learning rate parameter values, agent A3 will decide incorrectly when confronted with reward state-non-indicative negative reward observations, just as the heuristic agent A2. However, because the behavioral model of agent A2 has fewer model parameters compared to that of agent A3, its ensuing BIC values and PEPs were higher. Finally, for the highest learning rate parameter value η=2, the BIC value and PEP were maximal for the data analysis model based on agent A3. As discussed in the “Model Face Validity” section, a learning rate parameter of η=2 implies highly varying, non-converging behavior of the agent, which no longer aligns with the heuristic choice behavior of agent A2. Taken together, the model recovery results for agent A3 indicate that agent A3 cannot be well differentiated from agent A8 for small learning rate values and cannot be well differentiated from agent A2 for medium learning rate parameter values, respectively. Finally, note that while agent A4 can also mimic the behavior generated by agent A3 by adopting identical values for its two learning rate parameters, its inherent higher complexity (three instead of two free parameters) resulted in lower BIC values compared to agents A3 and A8. Our model comparison framework thus reliably differentiates between agents A3 and A4 when applied to data generated by agent A3.

For data generated based on agent A4, Figs. 6 and 7 visualize model recovery analyses results as a function of the negative reward learning rate parameter $\eta _n$ for a post-decision noise parameter τ=0.01 and a positive reward learning rate parameter of $\eta _p = 0.01$. Again, we focused on a low post-decision noise scenario and also note that from Fig. 5, the positive reward learning rate parameter $\eta _p$ affected the generated behavior only marginally. For low negative reward learning rate values of agent A4, the data analysis models based on agents A3 and A8 yielded the highest BIC values and highest PEPs. For medium negative reward learning rate parameters, the data analysis model based on agent A2 achieved the highest BIC values and highest PEPs. For high negative reward learning rate parameters, the BIC values and PEPs were maximal for the data analysis models based on agents A3 and A8. In the context of our model space and for the given generative parameter settings, agent A4 is thus not recoverable. This indicates that the behavior generated by the behavioral model of agent A4 can be mimicked by agents A2, A3, and A8, which can achieve similar behavior with fewer free parameters. Stated differently, the additional positive reward component learning rate parameter of agent A4 did not result in a unique data feature given the current task design.

For data generated based on agent A5, Figs. 6 and 7 depict model recovery analyses results as a function of the prior expectation bias parameter π for a post-decision noise parameter τ=0.01 and a positive reward learning rate parameter of $\eta _p = 0.01$. Again, we focused on a low post-decision noise scenario and also note that from Fig. 5, a low positive reward learning rate parameter $\eta _p$ yielded the most characteristic descriptive behavior. For all selected parameter space entries, the highest BIC values and highest PEPs were achieved by the data analysis model based on agent A5. Therefore, the results indicate that in the generative parameter setting considered, agent A5 is reliably recoverable.

For data generated based on agent A6, the highest BIC values and highest PEPs were achieved by the data analysis model based on agent A6 for all values of the post-decision noise parameter τ. Similarly to the behavioral models based on agents A1 and A2, the behavioral model of agent A6 is thus reliably recoverable, even in higher post-decision noise scenarios.

For data generated based on agent A7, Figs. 6 and 7 visualize model recovery analyses results as a function of the prior expectation bias parameter π in the low post-decision noise scenario of τ=0.01. For all selected parameter values, the BIC values and PEPs were maximal for the data analysis model based on agent A7, indicating reliable recovery of this agent model.

Finally, for data generated based on agent A8, Figs. 6 and 7 depict the model recovery analyses results as a function of the negative reward learning rate parameter $\eta _n$ in the low post-decision noise scenario of τ=0.01. In line with the results discussed above for models A3 and A4, for low and high negative reward learning rate parameter values, the BIC values and PEPs were maximal for the behavioral models of agents A3 and A8. For medium negative reward learning rate parameter values, the BIC values and PEPs were maximal for the model based on agent A2. These results corroborate the finding discussed above that agents A3, A4, and A8 cannot be well differentiated in the current experimental task.

#### Parameter Recovery

**Fig. 8:**
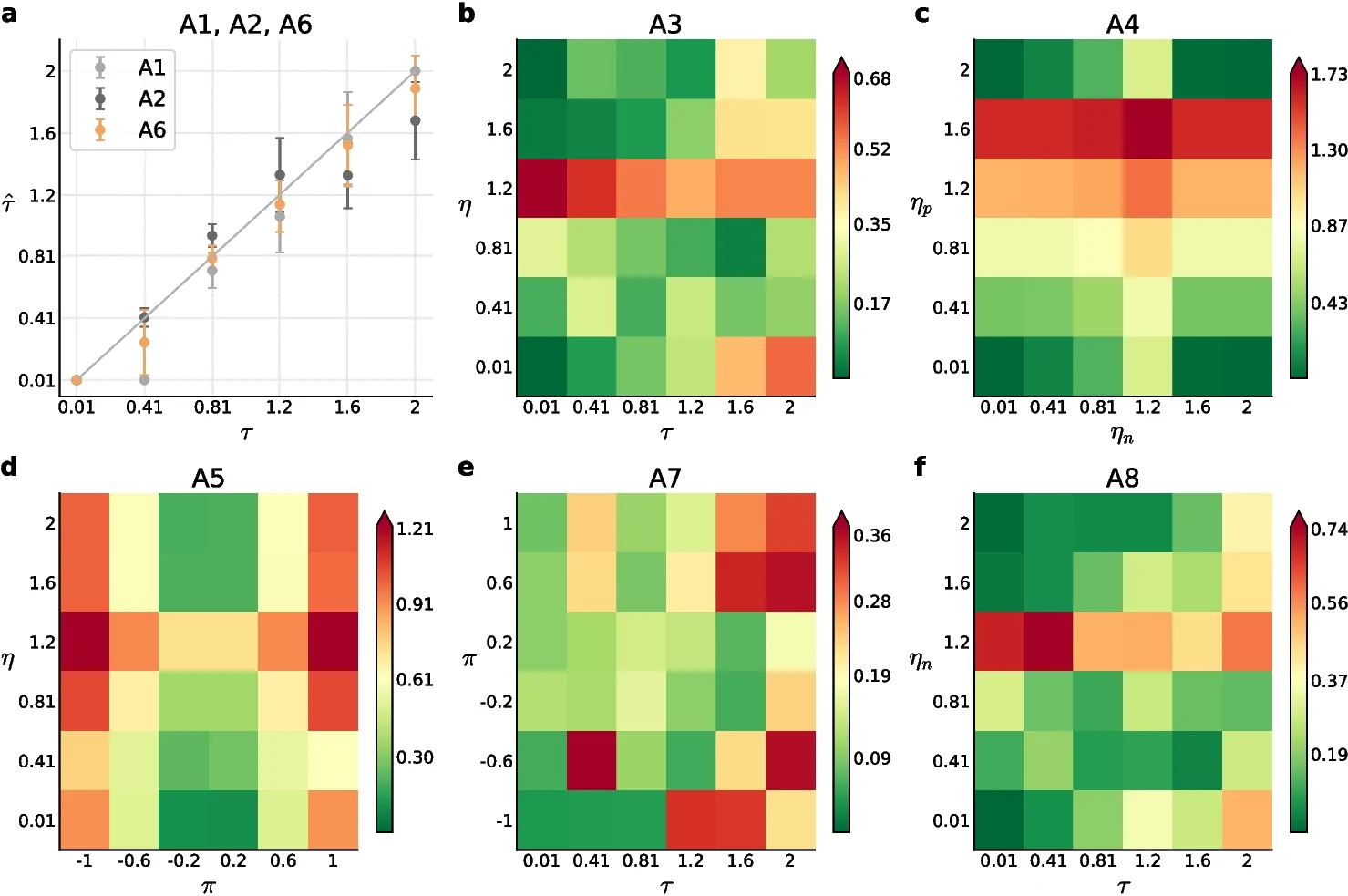
Parameter recovery results for all agents with free parameters. **a** The panel shows the ML estimates $\hat{\tau }$ as a function of the data-generating post-decision noise parameter τ. Agents A1, A2, and A6 are differentiated in terms of the colors (light gray, dark gray, orange). The diagonal line corresponds to perfect parameter estimation. Markers represent average ML estimates and whiskers refer to *SD*s across the artificially generated data sets. **b-f** Colors encode the Euclidean distance between the average ML estimates and the true data-generating parameter values. All parameter values were rounded to two decimal places. For the generation of this figure, please refer to *abm_figure_8.py*

To assess the degree to which we can reliably estimate the parameters of each behavioral model in our model space, we performed a series of parameter recovery analyses. Specifically, for a given data-generating model and parameter setting, we evaluated the ML estimates of the data-generating model across all artificially generated data sets using the procedures described in the “Parameter Estimation andModel Comparison” section. For one-dimensional parameter spaces, we evaluated the average ML estimates and the corresponding standard deviations for each parameter value. For two-dimensional parameter spaces, we evaluated the Euclidean distance between the average ML estimates and the true, data-generating parameter values as a measure of the discrepancy between the estimates and the true values. Figure 8 summarizes the results of these analyses.

Figure 8a shows the ML estimate $\hat{\tau }$ as a function of the data-generating parameter values τ of the behavioral models based on agents A1, A2, and A6. In general, ML estimates were consistent with their respective data-generating parameters for most values of τ. For $\tau \in \{0.41, 1.6, 2\}$, the true post-decision noise parameter values were slightly underestimated, especially for agent A1. These results are in line with the model face validity results (cf. “Model Face Valid-ity” section). Specifically, the behavior generated based on a post-decision noise parameter value of τ=0.41 was hardly distinguishable from the behavior generated based on τ=0.01. Moreover, for large post-decision noise parameter values, the behavioral data generated by any model converges to the data generated by a purely random choice policy strategy as implemented for agent A0 (Fig. 5a). Thus, the ML estimate for τ reaches an asymptote.

Figure 8b depicts the Euclidean distances between the average ML estimates and the true data-generating parameters for the behavioral model based on agent A3. In general, the closest alignment between average ML estimates and true data-generating parameters was achieved for low values of the post-decision noise parameter τ, while for higher values of τ, the ML estimates became less consistent with their corresponding true values. Concerning the learning rate parameter η, the ML estimates were most consistent with their true values for small and large values of η. However, for medium values η, the behavior generated by the behavioral model of agent A3 was not markedly different from that generated by a low value of η (Fig. 5b), reducing the identifiability of the data-generating parameters. Similarly, for data generated based on agent A4, the positive reward learning rate parameter $\eta _p$ could only be reliably recovered for very small or very large values due to the limited impact on the generated behavior. The ML estimates of the data-generating negative reward learning rate parameter $\eta _n$ were mostly consistent with the true parameter values with minor decreases in the medium value range (Fig. 8c).

For data generated based on the behavioral model of agent A5, Fig. 8d shows that its parameters could be recovered well for positive and negative values of the prior expectation bias parameter π close to zero and low and high values of the learning rate parameter η. Again, for medium values of η, the generated behavior did not differ sufficiently from that for low values of η, hampering parameter identifiability. For more extreme positive or negative values of π, the ML estimates were mostly inconsistent with the data-generating parameters due to the minor impact of the precise value of π on the behavior generated by this agent model.

Figure 8e shows that for data generated based on agent A7, parameters could be recovered relatively well, as indicated by the lower maximum value of the Euclidean distance when compared to the other agents with two- and three-dimensional parameter spaces. As expected, parameter recoverability is mainly affected by the post-decision noise parameter τ. Finally, in line with the results for agents A3, Fig. 8f shows that low and high negative reward learning rate parameter values could be reliably recovered for data generated based on the behavioral model of agent A8. Again, medium values of $\eta _n$ resulted in lower identifiability results due to the limited impact on the generated behavior. For the post-decision noise parameter τ, parameter recoverability again reduced with higher parameter values.

**Fig. 9:**
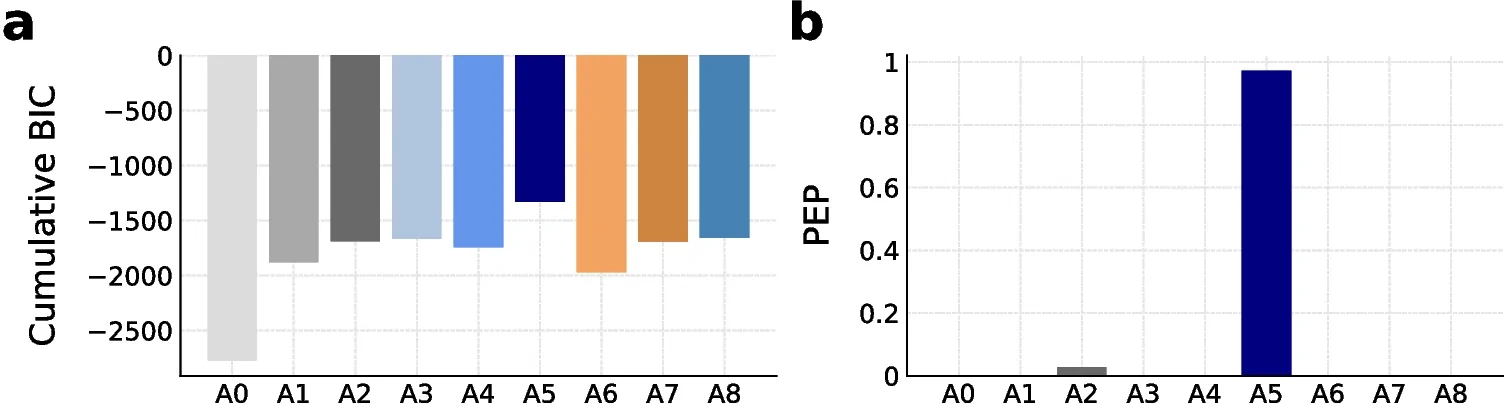
Evaluation of the behavioral models in light of the experimental data. **a** Group-cumulative BIC values (i.e., the sum of participant-specific BIC values) for each behavioral model. Behavioral models are differentiated in terms of the bar colors. Higher BIC values indicate higher model plausibility (cf. Schwarz, 1978). The highest group-cumulative BIC value was achieved by the behavioral model based on agent A5 (dark blue-colored bar). **b** PEPs for all behavioral models. Higher PEPs indicate higher model plausibility. The PEP was maximal for the behavioral model based on agent A5. For the generation of this figure, please refer to *abm_figure_9.py*

### Evaluation of the Experimental Data

Upon validating our behavioral models, we evaluated them in light of the experimental data to achieve a better understanding of the mechanisms underlying the experimentally observed behavior described in the “Descriptive Analyses” section. To this end, we applied the parameter estimation and model comparison procedures to the experimentally acquired 50 participant data sets. Figure 9 visualizes the results. The group-cumulative BIC value was maximal for agent A5 (Fig. 9a) and the PEP of agent A5 was approximately 0.97 (Fig. 9b). At the level of individual participants, the BIC value was maximal for the behavioral model based on agent A5 for 20 of the 50 participants, while the behavior of a group of 14 other participants could be most plausibly explained by the behavioral model based on agent A2. For this model, a PEP of 0.03 was observed.

The ML parameter estimates $\hat{\eta }$, $\hat{\pi }$, and $\hat{\tau }$ of the group-level winning model A5 varied significantly across the 50 participants. Specifically, the estimated learning rate parameter values $\hat{\eta }$ ranged from 0.01 to 1.56 with an average of 0.45 ± 0.07, the estimated prior expectation parameter values $\hat{\pi }$ ranged from -1 to 1 with an average of 0.31 ± 0.08, and the estimated post-decision noise parameter values $\hat{\tau }$ ranged from 0.01 to 2 with an average of 0.68 ± 0.04. As shown in Fig. 8d, these parameter estimates lie, on average, in an identifiable region of the model’s parameter space, which we also corroborated on an individual participant level using post-hoc model validation analyses as discussed below. Notably, the mostly positive valued parameter estimates for the prior expectation parameter are well in line with the descriptive finding that participants were biased away from deciding for high negative reward states and thus achieved low accuracy levels in particular for high negative reward states (cf. “Descriptive Analyses” section).

Taken together, the results indicate that agent A5 provides the most plausible explanation of the participants’ learning and reward state decision-making behavior among the set of behavioral models assessed. As the behavioral model based on agent A2 accounted best for the behavior of 14 participants, a subgroup of participants apparently did not learn the associations of visual search field patterns and reward states, but followed a heuristic decision strategy.

#### Post-Hoc Model Validation

**Fig. 10:**
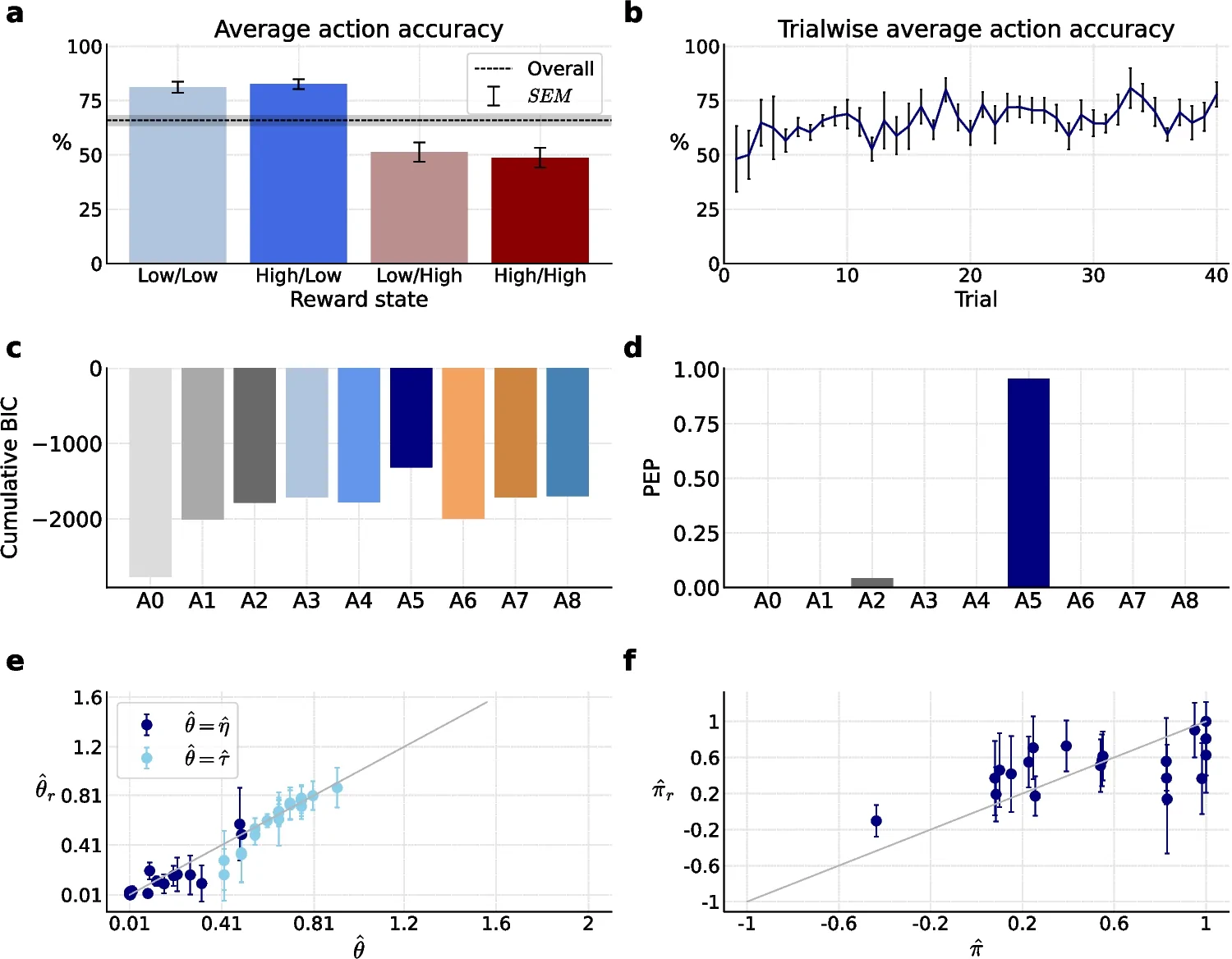
Post-hoc model validation analyses results. The panels depict post-hoc model validation analyses results of 50 data sets generated based on the most plausible behavioral model and the corresponding ML parameter estimates for each participant. The data analysis procedure was repeated 10 times. **a** Average action accuracy by reward state. The dashed line represents the overall average action accuracy across all reward states and the 10 data analysis repeats. The light gray area around this dashed line refers to the average *SEM*. The colored bars represent the reward state-specific average action accuracies. The error bars depict the *SEM*. **b** Trialwise average action accuracy across all reward states and the 10 data analysis repeats. Error bars refer to the *SD* across the 10 data analysis repeats. **c** Group-cumulative BIC values for all behavioral models in our model space. Behavioral models are differentiated by their bar colors. Higher BIC values refer to higher model plausibility (cf. Schwarz, 1978) **d** PEPs for all behavioral models in our model space. **e** Parameter recovery results for agent A5 (n=20). ML estimates of the experimental data are displayed on the x-axis, where $\hat{\theta }$ either refers to the estimated learning rate parameter values $\hat{\eta }$ or the estimated post-decision noise parameter values $\hat{\tau }$. The corresponding recovered average ML estimates $\hat{\theta }_r$ are displayed on the y-axis. Error bars refer to *SD*s across the 10 data analysis repeats. **f** Parameter recovery results for agent A5’s prior expectation bias parameter. The recovered average ML estimates $\hat{\pi }_r$ are shown as a function of the participant-specific ML estimates $\hat{\pi }$. For the generation of this figure, please refer to *abm_figure_10.py*

Upon identifying the most plausible behavioral model for the experimentally observed behavioral data of 50 participants at the group level, we performed post-hoc model validation analyses by generating simulated data sets based on the model comparison results reported in the “Evaluationof the Experimental Data” section. To this end, we simulated 50 participant data sets using the most plausible model of each participant and the corresponding ML parameter estimates. As in the “Model Validation” section, the simulated data sets comprised the agents’ actions on 40 experimental trials, with reward state, visual search field observation, and reward sequences corresponding to the actual participant-specific sequences. To minimize the effect of random action variation, we repeated this process 10 times and evaluated the average action accuracies across these data generation repeats. Specifically, we evaluated the reward state-specific and trialwise average action accuracies as for the participant data sets (see Fig. 2). Similarly, we subjected the generated data sets to the parameter estimation and model comparison procedure to evaluate the average model and parameter recoverability performance. Figure 10 displays the results.

The descriptive analyses of the post-hoc model validation results revealed an overall average action accuracy of 65.92% ± 2.46 across all reward states and data analysis repeats (Fig. 10a). The highest reward state-specific average action accuracy of 82.56% ± 2.28 was observed for the reward state *high positive reward/low negative reward*, closely followed by the reward state *low positive reward/low negative reward* with an average action accuracy of 81.12% ± 2.47. Similar to the experimental data, the average action accuracies were in general lower for the high negative reward states (*low positive reward/high negative reward*: 51.28% ± 4.39, *high positive reward/high negative reward*: 48.7% ± 4.54). Moreover, Fig. 10b shows that the trialwise average action accuracies increased on average from 48.2% (SD=15.1 across data analysis repeats) to 77.8% (SD=5.69 across data analysis repeats). In general, the descriptive analyses results of the post-hoc model validation align well with those of the experimental data, supporting the validity of our agent-based modeling approach.

As depicted in Fig. 10c, the model comparison procedure showed that the group-cumulative BIC value was maximal for the behavioral model based on agent A5. Similarly, the highest PEP of 0.96 was achieved by agent A5 (Fig. 10d). Moreover, the parameter recovery results confirmed that the estimated parameter values of the most plausible behavioral model based on agent A5 could largely be reliably recovered for those participants, for which this model explained the behavioral data best (Fig. 10e and f). Taken together, these results support the behavioral validity of our behavioral model set and the inferences drawn based on the experimental data in the “Evaluation of the Experimental Data” section.

## Discussion

In this work, we addressed the question of how humans learn associations between complex visual stimuli and latent reward states that are characterized by both positive and negative reward levels and induce concurrent approach-avoidance behavioral tendencies. First, by developing a novel translational approach-avoidance conflict paradigm, we demonstrated that human participants can learn the necessary associations and exhibit a potentially instruction-induced response bias for underestimating high negative reward states on early trials. Second, we developed a comprehensive Rescorla-Wagner and control agent-based behavioral model set. A series of model face validity and recovery analyses validated our initiative and supported the robustness and reliability of our model-based inferences. Third, we demonstrated that for the current task, human participants tend to apply Rescorla-Wagner learning-based strategies with a single learning rate parameter and a prior expectation bias parameter that accounts for the underestimation of high negative reward states. In the following, we discuss our three main contributions in more depth and highlight some limitations of our study along with some suggestions for future research.

As the first of our contributions, we introduced a novel approach-avoidance conflict paradigm that allowed us to exclusively investigate human learning processes in the context of approach-avoidance behavior acquisition. As such, the paradigm was inspired by translational approaches to AACs (Kirlic et al., 2017) and dynamic visual foraging tasks (Kristjánsson et al., 2020). In contrast to previous studies that employed computational methods for explaining AAC behavior, positive and negative reward stimuli were not associated with an action executed by the participant (e.g., selecting one option from two options) but with more general environmental context stimuli (i.e., visual search field patterns). In doing so, we aimed for a higher ecological validity of our results given that most real-life learning processes involve rather complex associations between differently valued stimuli and context characteristics (Lissek et al., 2006; Beckers et al., 2013). As a consequence and complementing previous computational modeling work in this area (e.g., Talmi et al., 2009; Bublatzky et al., 2017; Mkrtchian et al., 2017; Aylward et al., 2019; Aupperle et al., 2011; Moughrabi et al., 2022; Yamamori and Robinson, 2023), the modeled participant actions served as measures of the learning process and not as indicators of already acquired approach and avoidance behavioral tendencies.

Our second contribution concerns the formulation and validation of a set of agent-based behavioral models to achieve a better understanding of the key mechanisms underlying participants’ observed learning processes. Specifically, our model space included control and heuristic agents that modeled participants’ actions solely based on currently available information, being incapable of storing information across trials. Moreover, we considered different Rescorla-Wagner learning-based agents that based their decisions on the expected reward value estimates and integrated information across trials by sequentially updating their expectations according to the Rescorla-Wagner learning rule. Importantly, our Rescorla-Wagner learning-based agents differed in terms of their learning rate parameter(s) and the form of the prior expected reward value estimates. Finally, we included a hybrid agent that learned negative reward in a Rescorla-Wagner learning-based manner and decided on the level of positive reward based on the currently available information. The inclusion of the latter agent model was motivated by the design of the task. Specifically, the task design did not require learning processes for the positive reward component given its deterministic nature. Overall, we could demonstrate adequate recoverability of our model space, allowing us to draw valid model-based inferences.

As a third contribution, we showed that the most plausible model in light of the experimental data was a Rescorla-Wagner learning-based strategy that uses a single learning rate parameter, a prior expectation bias parameter, and a post-decision noise parameter. This result can be explained in terms of the deterministic nature of the positive reward component and the task instructions. Due to the design of the task, reward-specific learning rate parameters did not contribute to a better explanation of the observed behavior. Moreover, participants were instructed that a single observation of electrotactile stimulation would not be informative enough to decide on the level of negative reward. That is, if an electrotactile stimulation was first observed within the first trials, the associated latent reward state could correspond to both low and high negative reward. Interestingly, Mkrtchian et al. (2017) also showed that a paradigm-adapted Rescorla-Wagner learning-based model explained the trialwise Go/NoGo actions of their participants best. Similarly, research on avoidance learning in classical bandit tasks indicates that computational models that incorporate human learning biases in the form of additional learning rate parameters best describe the observed data (Palminteri et al., 2015; Palminteri and Lebreton, 2021; Vrizzi et al., 2023; Salem-Garcia et al., 2023). Consequently, there seems to be no single best Rescorla-Wagner learning-based agent, but rather a need for agent models that are designed based on the specific paradigm and task instructions (cf. Wolpert and Macready, 1997). This finding aligns well with the work by Eckstein et al. (2022) who recently showed that computational modeling parameters possess limited generalizability across tasks and therefore must be interpreted in a task-specific manner. It should also be noted that previous computational modeling studies commonly investigated the value of a reward or punishment sensitivity parameter, capturing subjective weighting processes of observed rewards and punishment (e.g., Mkrtchian et al., 2017; Aylward et al., 2019; Yamamori and Robinson, 2023). Introducing such sensitivity parameters in our Rescorla-Wagner learning-based agent models would have solely influenced the size of the observed positive and negative reward values but not the observed learning behavior itself, making such parameters barely recoverable, which is why we omitted them. Finally, it should be highlighted that previous studies also report on behavioral biases. For example, Pedersen et al. (2021) found that participants with major depressive disorder exhibited a bias away from the approach boundary of a drift diffusion model. Likewise, Mkrtchian et al. (2017) report on an overall bias towards go responses.

Despite the growing interest in the application of computational modeling to approach-avoidance conflict, only few studies have thus far considered learning processes in their task design and model specification. For example, studies by Pedersen et al. (2021), Smith et al. (2021), and Zorowitz et al. (2019) provided participants with visual information on reward and punishment probabilities, such that no learning was required. Moreover, if learning was considered previously, participants often faced task designs that required them to optimize their decisions while actively learning (e.g., bandit task designs; Aylward et al., 2019; Moughrabi et al., 2022). Hence, there was no clear separation of learning and decision-making processes, making it difficult to pinpoint causes of deficits since these might have occurred at both stages. In addition, there exist some studies at the intersection of approach-avoidance conflict and associative learning. For example, Chu et al. (2021) required participants to learn object-valence associations that were later combined with potentially rewarded approach-avoidance decisions. Avoidance behavior did not result in any adverse outcomes apart from the failure to earn additional points. Moreover, this study did not apply computational modeling to the learning phase. Finally, we would like to highlight that we here defined learning by means of trial-by-trial responses to contingency ratings and not in terms of choice behavior. In contrast, Yamamori et al. (2023), for example, defined learning in terms of the influence of previously observed outcomes on subsequent actions. In sum, the novelty and value of our study stem from its joint consideration of computational modeling, approach-avoidance conflict, and associative learning.

Last, we consider some limitations of our study along with some suggestions for future research. First, in contrast to previous AAC studies and for reasons of experimental simplification in an already complex paradigm, our task invoked a deterministic positive reward, thus inducing a level of asymmetry in the uncertainty associated with the positive and negative reward components. In particular, this characteristic of the task likely contributed to the finding of a single best learning rate parameter. Thus, it remains an open question whether this result can be replicated when using a probabilistic positive reward and, more generally, if the levels of uncertainty associated with positive and negative reward components bear on learning processes in AAC acquisition. Second, from a computational modeling perspective, the model and parameter recovery analyses results were not unanimously good. Specifically, agent models A3, A4, and A8 could not be well differentiated in terms of their generated actions. Moreover, parameter recovery was less reliable in higher-dimensional parameter spaces. This finding stresses the importance of paradigm-tailored models and model validation analyses when studying human behavior from a computational perspective (cf. Wilson and Collins, 2019; Letkiewicz et al., 2023). Third, due to the focus on introducing a comprehensive agent-based behavioral modeling framework in the context of avoidance learning, our work did not explore inter-individual differences in terms of, for instance, correlations between model-specific parameters (e.g., the learning rate parameter) and clinically relevant variables (e.g., state- and trait-anxiety). However, previous research reported higher negative reward learning rates and lower positive reward learning rates for anxious and depressed individuals (Aylward et al., 2019; Pike and Robinson, 2022). Moreover, there is evidence that task-induced anxiety is associated with lower learning rates (Yamamori and Robinson, 2023). Future research could therefore investigate such clinically relevant correlations and extend our current results by exploring the approach-avoidance behavior in the second phase of the experiment. Similarly, a future objective could be the incorporation of cognitive processes that have been shown to play a central role in learning paradigms, such as working memory (Collins et al., 2017; Yoo and Collins, 2022), in the agent-based modeling framework. Fourth, while both primary and secondary reinforcers are generally considered suitable for inducing conditioned responses, there is evidence that different neural systems may underlie the processing of monetary win/loss (more reliant on striatal regions) versus affective/pain-related outcomes such as electrotactile stimulation (more reliant on amygdala regions; Delgado et al., 2011). In future studies, it would therefore be interesting to investigate how different primary and secondary reinforcers influence the learning process. Fifth, despite the advanced complexity of our task, greater ecological validity could be achieved by implementing a similar task design in a virtual reality environment, as has been done, for example, in research on context conditioning (Grillon et al., 2006; Kroes et al., 2017). Finally, it should be noted that computational modeling results are highly dependent on task characteristics, making it difficult to compare the results across studies and to draw strong conclusions. Thus, future studies should aim for more similar task designs, samples, and overall study goals (Loijen et al., 2020; Letkiewicz et al., 2023).

To conclude, we here introduced a novel approach-avoidance conflict task that allowed us to exclusively investigate human learning processes in the overall context of avoidance behavior acquisition. Using a computational agent-based behavioral modeling approach, we provide some initial groundwork for a deeper and more fine-grained understanding of the learning processes resulting in AACs and thereby hope to contribute to the overarching aim of a better understanding of the etiology of anxiety disorders.

## Figures and Tables

**Fig. 11:**
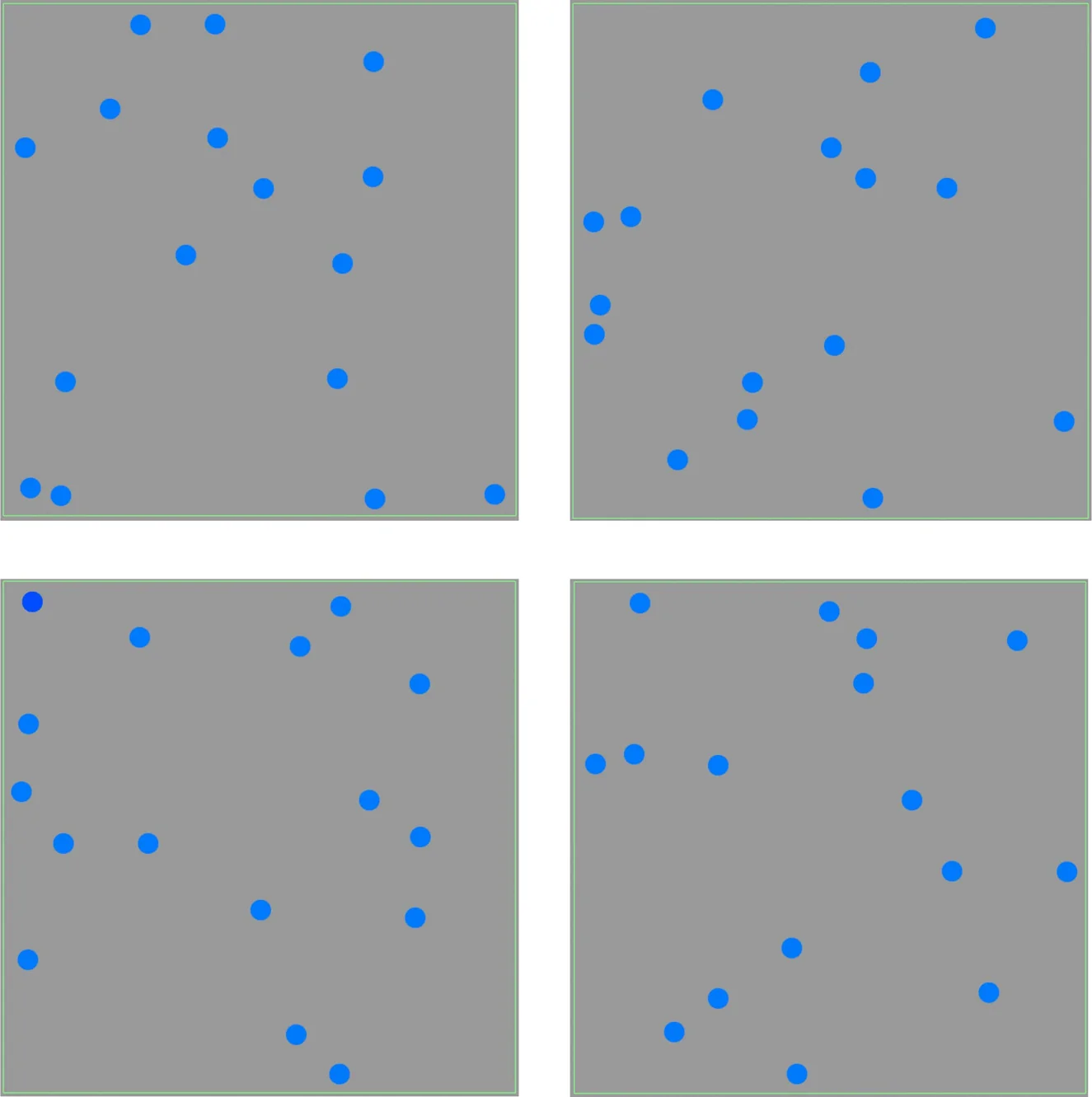
The four visual search field patterns used in the experiment

## Data Availability

The code, anonymized data, and study materials can be accessed at https://osf.io/bjdse/.

## Appendix A: Self-Report Data

Before receiving detailed instructions on the experiment, participants were asked to complete the following questionnaires measuring anxiety- and mood-related constructs: the Positive and Negative Affect Schedule (PANAS; Krohne et al. (1996)), the State-Trait Anxiety Inventory (trait anxiety, STAI-T; Laux et al. (1981)), the Anxiety Sensitivity Index-3 (ASI-3, Kemper et al. (2011)), and the BIS/BAS scales (Strobel et al., 2001).

After each experimental phase, participants were asked to answer the following questions for each of the four visual search fields presented and for four additional, not previously shown visual search fields, which served as distractor items.

1. Have you seen this search field? (yes/no)
2. How confident are you in your decision to have (not) seen this search field? (0 (not confident at all) - 100 (very confident))
3. How unpleasant do you find this search field? (0 (not unpleasant at all) - 100 (very unpleasant))
4. How pleasant do you find this search field? (0 (not pleasant at all) - 100 (very pleasant))
5. How calming or arousing do you find this search field? (0 (very calming) - 100 (very arousing))
6. How anxious did you feel when you searched for coins in this search field? (0 (not anxious at all) - 100 (very anxious))
7. When you searched for coins in this search field, how likely do you think it was that you would receive an electrotactile stimulation? (in %)
8. When you searched for coins in this search field, how many coins do you think could be found? (discrete number)

In addition, participants were asked the following two questions after each experimental phase.

1. Did you know in which search fields you would have a high probability of receiving an electrotactile stimulation? (yes/no)
2. Did you know in which search fields you would have a low probability of receiving an electrotactile stimulation? (yes/no)

At the end of the experiment, participants were again required to complete the PANAS and answer the following questions on unipolar scales, ranging from *Not at all* (0) to *Strongly* (100).

1. How much anxiety did you feel when you received electrotactile stimulation?
2. How much joy or pleasure did you feel when you found a coin?
3. How stressful did you find the experiment?
4. How difficult was it for you to make decisions during the experiment?
5. How motivated were you to collect coins?
6. How motivated were you to avoid electrotactile stimulation?
7. How pleasant was the experience of collecting a coin?
8. How unpleasant was the experience of receiving electrotactile stimulation?

Furthermore, participants were asked how attractive they consider the following scenarios on a bipolar scale, ranging from *very unattractive* (0) to *very attractive* (100).

1. I receive electrotactile stimulation with a probability of 10% and win 2 coins.
2. I receive electrotactile stimulation with a probability of 80% and win 2 coins.
3. I receive electrotactile stimulation with a probability of 10% and win 6 coins.
4. I receive electrotactile stimulation with a probability of 80% and win 6 coins.

## Appendix B: Experimental Task Instructions

### B.1 German Original

In addition to the instructions, a short video demonstrating the completion of exemplary trials was presented to participants (https://osf.io/bsg5c/, “Demonstrations.mp4”).

**Herzlich Willkommen zum Experiment!**

Vielen Dank, dass Sie sich bereiterklärt haben, an unserem Experiment teilzunehmen. Im Folgenden wird Ihnen der Ablauf des Experimentes erläutert. Bei Fragen wenden Sie sich bitte jederzeit an den/die Versuchsleiter/in.

Allgemeine Informationen zum Experiment

Mit diesem Experiment möchten wir menschliches Suchverhalten unter Bedrohung untersuchen. Dazu werden Sie auf 4 verschiedenen Suchfeldern goldfarbene Münzen suchen. Jedes Suchfeld wird durch eine spezifische Anordnung von 16 blauen Kreisen gekennzeichnet sein.

Unter einigen Kreisen werden sich die zu suchenden goldenen Münzen befinden. Mithilfe eines Mausklicks können Sie diese Maskierung aufheben und herausfinden, ob sich hinter dem Kreis eine Münze verbirgt. Befindet sich unter dem angeklickten Kreis eine Münze, so können Sie diese durch einen weiteren Mausklick einsammeln. Nach Einsammeln der Münze wird die Farbe des Kreises für einen kurzen Moment auf Rot wechseln. Dies bedeutet, dass sich nun keine Münze mehr hinter dem Kreis verbirgt. Durch das Einsammeln von Münzen erhöhen Sie Ihr Spielkonto, das Ihnen in Form eines Scores am oberen Bildschirmrand angezeigt werden wird. Während Sie sich in einem Suchfeld befinden, besteht das Risiko elektrotaktile Reize zu erhalten.

Beschreibung der Suchfelder

Die verschiedenen Suchfelder werden sich in **zwei Merkmalen** unterscheiden.

Einerseits werden die Suchfelder eine unterschiedliche Anzahl an Münzen aufweisen. So können in einem Suchfeld entweder **2** (niedriger Gewinn) oder **6** (hoher Gewinn) Münzen vorhanden sein. Die Position der Münzen, d.h. unter welchen Kreisen sie sich befinden, wird sich mit jeder Wiederholung verändern. Die Anzahl wird jedoch immer dieselbe sein.

Andererseits werden sich die Suchfelder hinsichtlich des Risikos, elektrotaktile Reize zu erhalten, unterscheiden. So kann das Risiko, elektrotaktile Reize in einem bestimmten Suchfeld zu erhalten, entweder niedrig oder hoch sein. Niedrig bedeutet, dass Sie in **1 der 10 Wiederholungen** eines Suchfeldes elektrotaktile Reize erhalten werden. Hoch bedeutet, dass Sie in **8 der 10 Wiederholungen** eines Suchfeldes elektrotaktile Reize erhalten werden.

Die folgende Tabelle veranschaulicht die beschriebenen Merkmale. Pro Merkmalskombination (z.B. niedriger Gewinn, niedriges Risiko) werden Sie ein Suchfeld kennenlernen und wiederholt durchlaufen.

Das Experiment besteht im Wesentlichen aus **zwei Phasen**.

Beschreibung der ersten Phase

Die erste Phase dient dazu, dass Sie sich mit den **4** verschiedenen Suchfeldern vertraut machen. Dazu werden Sie die einzelnen Felder jeweils **10 Mal** durchlaufen und in jedem Durchgang **alle** 16 Kreise einmal aufdecken.

Nach Aufdecken des 16. Kreises werden Sie automatisch zum nächsten Suchfeld weitergeleitet. Bitte beachten Sie, dass es für die zweite Phase des Experiments von Vorteil sein wird, wenn Sie sich merken, in welchen Suchfeldern eine niedrige bzw. hohe Anzahl an Münzen vorhanden ist und ob die Suchfelder mit einem niedrigen oder hohen Risiko, elektrotaktile Reize zu erhalten, einhergehen.

Zusammenfassung der ersten Phase des Experiments

- Sie werden 4 verschiedene Suchfelder kennenlernen.
- Jedes Suchfeld wird aus 16 verschieden angeordneten Kreisen bestehen, unter denen sich zum Teil Münzen befinden werden.
- Die Suchfelder werden sich in zwei Merkmalen unterscheiden:
  - Anzahl der zu suchenden Münzen,
  - Risiko, elektrotaktile Reize zu erhalten.
- Sie werden alle 16 Kreise durch einen Mausklick aufdecken müssen.
- Die Münzen gilt es durch einen weiteren Mausklick einzusammeln.
- Nach Anklicken aller 16 Kreise werden Sie automatisch zum nächsten Suchfeld weitergeleitet.

Beschreibung der zweiten Phase

In der zweiten Phase des Experiments werden Sie dieselben Suchfelder für weitere 10 Wiederholungen durchlaufen. Das Risiko, elektrotaktile Reize in einem Suchfeld zu erhalten, sowie die Anzahl der Münzen in einem bestimmten Suchfeld werden identisch zur vorherigen Phase sein. Auch in dieser Phase wird sich die Position, nicht aber die Anzahl, der Münzen über die Wiederholungen hinweg verändern.

Im Unterschied zur ersten Phase haben Sie in der zweiten Phase die Möglichkeit selbst zu entscheiden, wie lange Sie in einem bestimmten Feld suchen wollen. Es besteht folglich die Möglichkeit, den Erhalt von elektrotaktilen Reizen zu vermeiden, indem Sie das Suchfeld schnell verlassen. Sie werden zudem beliebig viele Kreise aufdecken dürfen. So können Sie sich zum Beispiel dazu entscheiden, gar keinen Kreis aufzudecken und das Suchfeld sofort zu verlassen, um das Risiko, elektrotaktile Reize zu erhalten, zu vermeiden. Gleichzeitig sollten Sie versuchen, möglichst viele Münzen einzusammeln, um Ihr Spielkonto (Score) zu erhöhen. Um ein präsentiertes Suchfeld zu verlassen, klicken Sie bitte auf das **EXIT-Feld** am unteren Bildschirmrand.

Zusammenfassung der zweiten Phase des Experiments

- Sie werden die 4 vertrauten Suchfelder erneut durchlaufen.
- Die Anzahl an Münzen sowie das Risiko, elektrotaktile Reize zu erhalten, werden für ein bestimmtes Suchfeld identisch zur ersten Phase sein.
- Sie werden selbst entscheiden, ob und wie lange Sie in einem Feld suchen wollen.
- Um zum nächsten Suchfeld zu gelangen, klicken Sie bitte auf das EXIT-Feld.

Start der ersten Phase

Nun beginnt die erste Phase des Experiments.

Bitte denken Sie daran, dass es in dieser Phase des Experiments darum geht, sich mit den Suchfeldern vertraut zu machen. Sie müssen hierbei in jedem Durchgang alle 16 Kreise einmal aufdecken. Nach jedem Durchgang werden Sie um Ihre Einschätzung der Merkmale des Suchfeldes gebeten. Sie werden hierzu die zuvor gezeigte Tabelle sehen und dazu aufgefordert, das präsentierte Suchfeld in einer der 4 möglichen Merkmalskombinationen einzuordnen (z.B. hoher Gewinn, hohes Risiko). Bitte bedenken Sie, dass Sie das Risiko, elektrotaktile Reize zu erhalten, erst nach mehreren Durchgängen korrekt einschätzen werden können.

Start der zweiten Phase

Nun beginnt die zweite Phase des Experiments.

Sie werden gleich dieselben Suchfelder für weitere 10 Wiederholungen durchlaufen. Bitte denken Sie daran, dass Sie nun beliebig viele Kreise aufdecken dürfen. Sie können die Suchfelder zudem zu jedem Zeitpunkt über das EXIT-Feld am unteren Bildschirmrand verlassen. Sie werden nicht automatisch zum nächsten Suchfeld weitergeleitet, sondern müssen selbst auf EXIT klicken. Sie haben dadurch die Möglichkeit, den Erhalt von elektrotaktilen Reizen zu vermeiden. Um zu entscheiden, ob und wie lange Sie in einem bestimmten Feld suchen wollen, können Sie Ihr Wissen aus der ersten Phase des Experiments nutzen. So werden die Anzahl an Münzen sowie das Risiko, elektrotaktile Reize in einem bestimmten Suchfeld zu erhalten, identisch zur ersten Phase sein.

Fast geschafft...

In den folgenden Durchgängen werden Sie die 4 vertrauten Suchfelder ein letztes Mal durchlaufen. Jedes Suchfeld wird Ihnen dabei **3 Mal** präsentiert werden. Anders als zuvor besteht nun **kein Risiko** mehr, elektrotaktile Reize zu erhalten. Identisch zur zweiten Phase des Experiments können Sie selbst entscheiden, wann Sie ein Suchfeld verlassen möchten. Um zum nächsten Suchfeld zu gelangen, müssen Sie auch hierbei auf das **EXIT-Feld** klicken.

### B.2 English Translation

**Welcome to the experiment!**

Thank you for agreeing to participate in our experiment. The procedure of the experiment is explained below. If you have any questions, please contact the experimenter at any time.

General information on the experiment

In this experiment, we aim to investigate human search behavior under threat. You will search for golden coins on 4 different search fields. Each search field will be characterized by a specific arrangement of 16 blue circles.

The golden coins you are looking for will be located under some of the circles. By clicking on the mouse, you can remove this masking and determine whether a coin is hidden below the circle. If there is a coin under the circle you clicked, you can collect it with another mouse click. After collecting the coin, the color of the circle will change to red for a moment. This means that there is no longer a coin below the circle. Collecting coins increases your score, which will be displayed at the top of the screen. While foraging in a search field, there is a risk of receiving electrotactile stimulation.

Description of the search fields

The various search fields will differ in **two characteristics**.

On the one hand, the search fields will comprise different numbers of coins. Specifically, there can be either **2** (low profit) or **6** (high profit) coins in a search field. The position of the coins, i.e., under which circles they are located, will change with each repetition. However, the number will always be the same.

On the other hand, the search fields will differ in terms of the risk of receiving electrotactile stimulation. Specifically, the risk of receiving electrotactile stimulation in a particular search field can be low or high. Low means that you will receive electrotactile stimulation in **1 of the 10 repetitions** of a search field. High means that you will receive electrotactile stimulation in **8 of the 10 repetitions** of a search field.

The following table illustrates the characteristics of the search fields. For each combination of characteristics (e.g., low profit, low risk), you will encounter one search field and run through it repeatedly.

The experiment consists basically of **two phases**.

Description of the first phase

The first phase is used to familiarize yourself with the **4** different search fields. To do this, you will go through the individual fields **10 times** and uncover **all** 16 circles once in each round.

After clicking on the 16th circle, you will automatically be forwarded to the next search field. Please note that it will be advantageous for the second phase of the experiment if you remember in which search fields there is a low or high number of coins and whether the search fields are associated with a low or high risk of receiving electrotactile stimulation.

Summary of the first phase of the experiment

- You will encounter 4 different search fields.
- Each search field will consist of 16 differently arranged circles, some of which will contain coins.
- The search fields will differ in two characteristics:
  - Number of coins to be searched for,
  - Risk of receiving electrotactile stimulation.
- You will have to reveal all 16 circles by clicking the mouse once.
- Coins can be collected by clicking the mouse again.
- After clicking on all 16 circles, you will automatically be forwarded to the next search field.

Description of the second phase

In the second phase of the experiment, you will go through the same search fields for another 10 repetitions. The risk of receiving electrotactile stimulation in a search field and the number of coins in a particular search field will be identical to that of the previous phase. Also in this phase, the location, but not the number, of coins will change over repetitions.

In contrast to the first phase, you can decide for yourself how long you want to search in a particular field. Therefore, it is possible to avoid receiving electrotactile stimulation by leaving the search field quickly. You will also be allowed to uncover as many circles as you wish. For example, you can decide not to uncover any circles at all and leave the search field immediately to avoid the risk of receiving electrotactile stimulation. At the same time, you should try to collect as many coins as possible to increase your total score. To exit a presented search field, please click on the **EXIT field** at the bottom of the screen.

Summary of the second phase of the experiment

- You will run through the 4 familiar search fields again.
- The number of coins and the risk of receiving electrotactile stimulation will be identical to the first phase for a particular search field.
- You will decide for yourself whether and for how long you want to search in a field.
- To proceed to the next search field, please click on the EXIT field.

Start of the first phase

The first phase of the experiment starts now.

Recall that the aim of this phase of the experiment is to familiarize yourself with the search fields. You must uncover all 16 circles once in each round. After each round, you will be asked to assess the characteristics of the search field. You will see the table shown above and will be asked to classify the search field presented in one of the 4 possible combinations of characteristics (e.g., high profit, high risk). Please note that you will only be able to correctly assess the risk of receiving electrotactile stimulation after several rounds.

Start of the second phase

The second phase of the experiment starts now.

You will immediately go through the same search fields for another 10 repetitions. Recall that now you can uncover as many circles as you wish. You can also exit the search fields at any time using the EXIT field at the bottom of the screen. You will not be automatically forwarded to the next search field, but you must click on EXIT yourself. This gives you the opportunity to avoid receiving electrotactile stimulation. To decide whether and for how long you want to search in a particular field, you can use your knowledge from the first phase of the experiment. The number of coins and the risk of receiving electrotactile stimulation in a particular search field will be identical to the first phase.

Almost there...

In the following rounds, you will go through the **4** familiar search fields one last time. Each search field will be presented to you **3 times**. Unlike before, there is **no longer any risk** of receiving electrotactile stimulation. Like in the second phase of the experiment, you can decide for yourself at which point you want to leave a search field. To move on to the next search field, you must also click on the **EXIT field**.

## Appendix C: Notational Remarks

*Variables and functions* In general, we denote elements of sets with lower-case letters, such as reward states s∈S, observations o∈O, rewards r∈R, expected reward value estimates $\hat{\mu } \in M$, decisions d∈D, and actions a∈A. When conceived as trial-by-trial variables, we equip the respective letter with a *t* subscript, such that for example $s_t = 0$ means that the reward state variable takes the value 0 on trial *t*. We denote functions in the general form

C1 $$\begin{aligned} f : D \rightarrow R, x \mapsto f(x), \end{aligned}$$

where *f* is the function label, *D* is the function’s domain, *R* is the function’s range, *x* is the function’s argument, and *f*(*x*) is a value of the function. We denote case-by-case definitions of a function’s values by

C2 $$\begin{aligned} f(x) := {\left\{ \begin{array}{ll} y & (x = a) \\ z & (x = b) \end{array}\right. }, \end{aligned}$$

meaning that *f* takes on the values f(a):=y and f(b):=z. ∧ denotes the logical AND.

*Vectors and matrices* We denote an *n*-dimensional vector of all ones by $1_n$, such that, for example, $1_4:= (1,1,1,1)^T$. We denote the *n*-dimensional canonical unit vector by $e_i, 1 \le i \le n$, such that, for example, $e_4:= (0,0,0,1)^T$. We denote the Kronecker product of two matrices $A \in \mathbb {R}^{m \times n}$ and $B \in \mathbb {R}^{p \times q}$ by ⊗, i.e.,

C3 $$\begin{aligned} A \otimes B = \left( \begin{array}{clc} a_{11} B & \cdots & a_{1n}B \\ \vdots & \ddots & \vdots \\ a_{m1} B & \cdots & a_{mn}B \\ \end{array}\right) \in \mathbb {R}^{pm \times qn}, \end{aligned}$$

such that, for instance,

C4 $$\begin{aligned} \left( \begin{array}{l} 0 \\ 1 \\ 0 \\ 0 \end{array}\right) \otimes \left( \begin{array}{l} r^p \\ r^n \end{array}\right) = \left( \begin{array}{l} 0\cdot r^p \\ 0\cdot r^n \\ 1\cdot r^p \\ 1\cdot r^n \\ 0\cdot r^p \\ 0\cdot r^n \\ 0\cdot r^p \\ 0\cdot r^n \\ \end{array}\right) = \left( \begin{array}{l} 0 \\ 0 \\ r^p \\ r^n \\ 0 \\ 0 \\ 0 \\ 0 \\ \end{array}\right) . \end{aligned}$$

We denote the Hadamard product of two matrices $A \in \mathbb {R}^{m \times n}$ and $B \in \mathbb {R}^{m \times n}$ by ∘, i.e.,

C5 $$\begin{aligned} A \circ B = \left( \begin{array}{clc} a_{11}b_{11} & \cdots & a_{1n}b_{1n} \\ \vdots & \ddots & \vdots \\ a_{m1}b_{m1} & \cdots & a_{mn}b_{mn} \\ \end{array}\right) \in \mathbb {R}^{m \times n}, \end{aligned}$$

such that, for instance,

C6 $$\begin{aligned} \left( \begin{array}{l} 0 \\ 0 \\ 1 \\ 1 \\ 0 \\ 0 \\ 0 \\ 0 \\ \end{array}\right) \circ \left( \begin{array}{l} x_1 \\ x_2 \\ x_3 \\ x_4 \\ x_5 \\ x_6 \\ x_7 \\ x_8 \\ \end{array}\right) = \left( \begin{array}{l} 0\cdot x_1 \\ 0\cdot x_2 \\ 1\cdot x_3 \\ 1\cdot x_4 \\ 0\cdot x_5 \\ 0\cdot x_6 \\ 0\cdot x_7 \\ 0\cdot x_8 \\ \end{array}\right) = \left( \begin{array}{l} 0 \\ 0 \\ x_3 \\ x_4 \\ 0 \\ 0 \\ 0 \\ 0 \\ \end{array}\right) . \end{aligned}$$

## Appendix D: Visual Search Field Patterns

Figure 11 displayse the four visual search field pattern stimuli used in the experiment. These specific stimuli were selected from 100 randomly generated proposal stimuli according to two main criteria. First, each quadrant of a visual search field had to include at least three circles. Second, no more than two circles were allowed to be arranged directly next to each other in a row (horizontally or vertically). For each participant in the experiment, the visual search field pattern stimuli were assigned a random observation number $o \in \{0,1,2,3\}$ based on a uniform random sampling procedure without replacement and visually presented at a screen size of approximately 22 × 22 cm with a resolution of 720 × 20 pixels.

## Appendix E: Model Face Validity Examples

### E.1 Agent A3

To illustrate the effect of the learning rate parameter η on the decision accuracy of agent A3, we consider the expected reward value estimate updates and the subsequent decisions for an exemplary sequence of trials with reward state s=3 (high positive reward, high negative reward) and thus visual search field observation o=3 (cf. (2)). Note that according to the definitions of (15), (16), and (20), for a given observation *o*, the expected reward value estimate updates and ensuing decisions are independent of the expected reward value estimate updates and ensuing decisions of the other three observations. For o=3, it thus suffices to consider the visual search field pattern observation-specific entries $\hat{\mu }^{o=3}_{r^p}$ and $\hat{\mu }^{o=3}_{r^n}$ in terms of their Rescorla-Wagner learning rule updates and as the basis for the agent’s decision function evaluation. Further note that according to the definition of the reward state-dependent reward distribution (3) as well as the veridical representation of this distribution in terms of the experimentally delivered stimuli (cf. “Experimental Design” section), the ten experimental trials corresponding to reward state s=3 comprise eight presentations of the reward state-indicative reward $r = (+1,-1)^T$ and two presentations of the reward state-non-indicative reward $r = (+1,+1)^T$. In the following, we thus consider the agent’s learning and decision-making-dependence for a small (η=0.01), medium (η=1.2), and large (η=2) learning rate for a fixed sequence of rewards, for which the reward state-non-indicative rewards appear on the second and fifth trial of the first five trials corresponding to s=3.

**Small learning rate parameter** η=0.01

Trial t=1, reward state-indicative reward $r_1 = (1,-1)^T$

$$\begin{aligned} \left( \begin{array}{r} \hat{\mu }^{o=3}_{{r_p}} \\ \hat{\mu }^{o=3}_{{r_n}} \\ \end{array}\right) _{1}= &  \left( \begin{array}{r} 0.0000 \\ 0.0000 \end{array}\right) + 0.01 \left( \left( \begin{array}{r} 1 \\ -1 \\ \end{array}\right) - \left( \begin{array}{r} 0.0000 \\ 0.0000 \\ \end{array}\right) \right) \\= &  \left( \begin{array}{r} 0.0000 \\ 0.0000 \\ \end{array}\right) + 0.01 \left( \begin{array}{r} 1.0000 \\ -1.0000 \\ \end{array}\right) \\= &  \left( \begin{array}{r} 0.0000 \\ 0.0000 \\ \end{array}\right) + \left( \begin{array}{r} 0.0100 \\ -0.0100 \\ \end{array}\right) \nonumber \\= &  \left( \begin{array}{r} 0.0100 \\ -0.0100 \\ \end{array}\right) \end{aligned}$$

$\Rightarrow \hat{\mu }_{r^p}^{o=3} \ge 0 \wedge \hat{\mu }_{r^n}^{o=3} < 0$ $\Rightarrow \phi (3) = 2 \wedge \phi (d) = 0$ for d≠3 $\Rightarrow \delta (\phi ) = 3$ ⇒ Correct decision d=3.

Trial t=2, reward state-non-indicative reward $r_2 = (1,1)^T$

$$\begin{aligned} \left( \begin{array}{r} \hat{\mu }^{o=3}_{{r_p}} \\ \hat{\mu }^{o=3}_{{r_n}} \\ \end{array}\right) _{2}= &  \left( \begin{array}{r} 0.0100 \\ -0.0100 \end{array}\right) + 0.01 \left( \left( \begin{array}{r} 1 \\ 1 \\ \end{array}\right) - \left( \begin{array}{r} 0.0100 \\ -0.0100 \\ \end{array}\right) \right) \nonumber \\= &  \left( \begin{array}{r} 0.0100 \\ -0.0100 \\ \end{array}\right) + 0.01 \left( \begin{array}{r} 0.9900 \\ 1.0100 \\ \end{array}\right) \\= &  \left( \begin{array}{r} 0.0100 \\ -0.0100 \\ \end{array}\right) + \left( \begin{array}{r} 0.0099 \\ 0.0101 \\ \end{array}\right) \nonumber \\= &  \left( \begin{array}{r} 0.0199 \\ 0.0001 \\ \end{array}\right) \end{aligned}$$

$\Rightarrow \hat{\mu }_{r^p}^{o=3} \ge 0 \wedge \hat{\mu }_{r^n}^{o=3} \ge 0$ $\Rightarrow \phi (1) = 2 \wedge \phi (d) = 0$ for d≠1 $\Rightarrow \delta (\phi ) = 1$ ⇒ Incorrect decision d=1.

Trial t=3, reward state-indicative reward $r_3 = (1,-1)^T$

$$\begin{aligned} \left( \begin{array}{r} \hat{\mu }^{o=3}_{{r_p}} \\ \hat{\mu }^{o=3}_{{r_n}} \\ \end{array}\right) _{3}= &  \left( \begin{array}{r} 0.0199 \\ 0.0001 \end{array}\right) + 0.01 \left( \left( \begin{array}{r} 1 \\ -1 \\ \end{array}\right) - \left( \begin{array}{r} 0.0199 \\ 0.0001 \\ \end{array}\right) \right) \nonumber \\= &  \left( \begin{array}{r} 0.0199 \\ 0.0001 \\ \end{array}\right) + 0.01 \left( \begin{array}{r} 0.9801 \\ -1.0001 \\ \end{array}\right) \\= &  \left( \begin{array}{r} 0.0199 \\ 0.0001 \\ \end{array}\right) + \left( \begin{array}{r} 0.0098 \\ -0.0100 \\ \end{array}\right) \nonumber \\= &  \left( \begin{array}{r} 0.0297 \\ -0.0099 \\ \end{array}\right) \end{aligned}$$

$\Rightarrow \hat{\mu }_{r^p}^{o=3} \ge 0 \wedge \hat{\mu }_{r^n}^{o=3} < 0$ $\Rightarrow \phi (3) = 2 \wedge \phi (d) = 0$ for d≠3 $\Rightarrow \delta (\phi ) = 3$ ⇒ Correct decision d=3.

Trial t=4, reward state-indicative reward $r_4 = (1,-1)^T$

$$\begin{aligned} \left( \begin{array}{r} \hat{\mu }^{o=3}_{{r_p}} \\ \hat{\mu }^{o=3}_{{r_n}} \\ \end{array}\right) _{4}= &  \left( \begin{array}{r} 0.0297 \\ -0.0099 \end{array}\right) + 0.01 \left( \left( \begin{array}{r} 1 \\ -1 \\ \end{array}\right) - \left( \begin{array}{r} 0.0297 \\ -0.0099 \\ \end{array}\right) \right) \nonumber \\= &  \left( \begin{array}{r} 0.0297 \\ -0.0099 \\ \end{array}\right) + 0.01 \left( \begin{array}{r} 0.9703 \\ -0.9901 \\ \end{array}\right) \\= &  \left( \begin{array}{r} 0.0297 \\ -0.0099 \\ \end{array}\right) + \left( \begin{array}{r} 0.0097 \\ -0.0099 \\ \end{array}\right) \nonumber \\= &  \left( \begin{array}{r} 0.0394 \\ -0.0198\\ \end{array}\right) \end{aligned}$$

$\Rightarrow \hat{\mu }_{r^p}^{o=3} \ge 0 \wedge \hat{\mu }_{r^n}^{o=3} < 0$ $\Rightarrow \phi (3) = 2 \wedge \phi (d) = 0$ for d≠3 $\Rightarrow \delta (\phi ) = 3$ ⇒ Correct decision d=3.

Trial t=5, reward state-non-indicative reward $r_5 = (1,1)^T$

$$\begin{aligned} \left( \begin{array}{r} \hat{\mu }^{o=3}_{{r_p}} \\ \hat{\mu }^{o=3}_{{r_n}} \\ \end{array}\right) _{5}= &  \left( \begin{array}{r} 0.0394 \\ -0.0198 \end{array}\right) + 0.01 \left( \left( \begin{array}{r} 1 \\ 1 \\ \end{array}\right) - \left( \begin{array}{r} 0.0394 \\ -0.0198 \\ \end{array}\right) \right) \\= &  \left( \begin{array}{r} 0.0394 \\ -0.0198 \\ \end{array}\right) + 0.01 \left( \begin{array}{r} 0.9696 \\ -1.0198 \\ \end{array}\right) \\= &  \left( \begin{array}{r} 0.0394 \\ -0.0198 \\ \end{array}\right) + \left( \begin{array}{r} 0.0096 \\ -0.0102 \\ \end{array}\right) \nonumber \\= &  \left( \begin{array}{r} 0.0490 \\ -0.0096 \\ \end{array}\right) \end{aligned}$$

$\Rightarrow \hat{\mu }_{r^p}^{o=3} \ge 0 \wedge \hat{\mu }_{r^n}^{o=3} < 0$ $\Rightarrow \phi (3) = 2 \wedge \phi (d) = 0$ for d≠3 $\Rightarrow \delta (\phi ) = 3$ ⇒ Correct decision d=3.

We thus observe that for the learning rate parameter and the scenario considered, the agent makes correct decisions for all reward state-indicative rewards and does not necessarily make an incorrect decision for a reward state-non-indicative reward.

**Medium learning rate parameter** η=1.2

Trial t=1, reward state-indicative reward $r_1 = (1,-1)^T$

$$\begin{aligned} \left( \begin{array}{r} \hat{\mu }^{o=3}_{{r_p}} \\ \hat{\mu }^{o=3}_{{r_n}} \\ \end{array}\right) _{1}= &  \left( \begin{array}{r} 0.0000 \\ 0.0000 \end{array}\right) + 1.2 \left( \left( \begin{array}{r} 1 \\ -1 \\ \end{array}\right) - \left( \begin{array}{r} 0.0000 \\ 0.0000 \\ \end{array}\right) \right) \\= &  \left( \begin{array}{r} 0.0000 \\ 0.0000 \end{array}\right) + 1.2 \left( \begin{array}{r} 1.0000 \\ -1.0000 \\ \end{array}\right) \\= &  \left( \begin{array}{r} 0.0000 \\ 0.0000 \end{array}\right) + \left( \begin{array}{r} 1.200 \\ -1.200 \\ \end{array}\right) \nonumber \\= &  \left( \begin{array}{r} 1.200 \\ -1.200 \\ \end{array}\right) \end{aligned}$$

$\Rightarrow \hat{\mu }_{r^p}^{o=3} \ge 0 \wedge \hat{\mu }_{r^n}^{o=3} < 0$ $\Rightarrow \phi (3) = 2 \wedge \phi (d) = 0$ for d≠3 $\Rightarrow \delta (\phi ) = 3$ ⇒ Correct decision d=3.

Trial t=2, reward state-non-indicative reward $r_2 = (1,1)^T$

$$\begin{aligned} \left( \begin{array}{r} \hat{\mu }^{o=3}_{{r_p}} \\ \hat{\mu }^{o=3}_{{r_n}} \\ \end{array}\right) _{2} =&\left( \begin{array}{r} 1.200 \\ -1.200 \\ \end{array}\right) + 1.2 \left( \left( \begin{array}{r} 1 \\ -1 \\ \end{array}\right) - \left( \begin{array}{r} 1.200 \\ -1.200 \\ \end{array}\right) \right) \\ =&\left( \begin{array}{r} 1.200 \\ -1.200 \\ \end{array}\right) + 1.2 \left( \begin{array}{r} -0.2200 \\ 2.2000 \\ \end{array}\right) \\ =&\left( \begin{array}{r} 1.200 \\ -1.200 \\ \end{array}\right) + \left( \begin{array}{r} -0.2400 \\ 2.6400 \\ \end{array}\right) \nonumber \\ =&\left( \begin{array}{r} 0.9600 \\ 1.4400 \\ \end{array}\right) \end{aligned}$$

$\Rightarrow \hat{\mu }_{r^p}^{o=3} \ge 0 \wedge \hat{\mu }_{r^n}^{o=3} \ge 0$ $\Rightarrow \phi (1) = 2 \wedge \phi (d) = 0$ for d≠1 $\Rightarrow \delta (\phi ) = 1$ ⇒ Incorrect decision d=1.

Trial t=3, reward state-indicative reward $r_3 = (1,-1)^T$

$$\begin{aligned} \left( \begin{array}{r} \hat{\mu }^{o=3}_{{r_p}} \\ \hat{\mu }^{o=3}_{{r_n}} \\ \end{array}\right) _{3} =&\left( \begin{array}{r} 0.9600 \\ 1.4400 \\ \end{array}\right) + 1.2 \left( \left( \begin{array}{r} 1 \\ -1 \\ \end{array}\right) - \left( \begin{array}{r} 0.9600 \\ 1.4400 \\ \end{array}\right) \right) \\ =&\left( \begin{array}{r} 0.9600 \\ 1.4400 \\ \end{array}\right) + 1.2 \left( \begin{array}{r} 0.0400 \\ -2.4400 \\ \end{array}\right) \\ =&\left( \begin{array}{r} 0.9600 \\ 1.4400 \\ \end{array}\right) + \left( \begin{array}{r} 0.0480 \\ -2.9280 \\ \end{array}\right) \nonumber \\ =&\left( \begin{array}{r} 1.0080 \\ -1.4480 \\ \end{array}\right) \end{aligned}$$

$\Rightarrow \hat{\mu }_{r^p}^{o=3} \ge 0 \wedge \hat{\mu }_{r^n}^{o=3} < 0$ $\Rightarrow \phi (3) = 2 \wedge \phi (d) = 0$ for d≠3 $\Rightarrow \delta (\phi ) = 3$ ⇒ Correct decision d=3.

Trial t=4, reward state-indicative reward $r_4 = (1,-1)^T$

$$\begin{aligned} \left( \begin{array}{r} \hat{\mu }^{o=3}_{{r_p}} \\ \hat{\mu }^{o=3}_{{r_n}} \\ \end{array}\right) _{4} =&\left( \begin{array}{r} 1.0080 \\ -1.4880 \\ \end{array}\right) + 1.2 \left( \left( \begin{array}{r} 1 \\ -1 \\ \end{array}\right) - \left( \begin{array}{r} 1.0080 \\ -1.4880 \\ \end{array}\right) \right) \\ =&\left( \begin{array}{r} 1.0080 \\ -1.4880 \\ \end{array}\right) + 1.2 \left( \begin{array}{r} -0.0800 \\ 0.4880 \\ \end{array}\right) \\ =&\left( \begin{array}{r} 1.0080 \\ -1.4880 \\ \end{array}\right) + \left( \begin{array}{r} -0.0096 \\ 0.5856 \\ \end{array}\right) \nonumber \\ =&\left( \begin{array}{r} 0.9984 \\ -0.9024 \\ \end{array}\right) \end{aligned}$$

$\Rightarrow \hat{\mu }_{r^p}^{o=3} \ge 0 \wedge \hat{\mu }_{r^n}^{o=3} < 0$ $\Rightarrow \phi (3) = 2 \wedge \phi (d) = 0$ for d≠3 $\Rightarrow \delta (\phi ) = 3$ ⇒ Correct decision d=3.

Trial t=5, reward state-non-indicative reward $r_5 = (1,1)^T$

$$\begin{aligned} \left( \begin{array}{r} \hat{\mu }^{o=3}_{{r_p}} \\ \hat{\mu }^{o=3}_{{r_n}} \\ \end{array}\right) _{5} =&\left( \begin{array}{r} 0.9984 \\ -0.9024 \\ \end{array}\right) + 1.2 \left( \left( \begin{array}{r} 1 \\ 1 \\ \end{array}\right) - \left( \begin{array}{r} 0.9984 \\ -0.9024 \\ \end{array}\right) \right) \\ =&\left( \begin{array}{r} 0.9984 \\ -0.9024 \\ \end{array}\right) + 1.2 \left( \begin{array}{r} 0.0016 \\ 1.9024 \\ \end{array}\right) \\ =&\left( \begin{array}{r} 0.9984 \\ -0.9024 \\ \end{array}\right) + \left( \begin{array}{r} 0.0019 \\ 2.2829 \\ \end{array}\right) \\ =&\left( \begin{array}{r} 1.0003 \\ 1.3805 \\ \end{array}\right) \end{aligned}$$

$\Rightarrow \hat{\mu }_{r^p}^{o=3} \ge 0 \wedge \hat{\mu }_{r^n}^{o=3} \ge 0$ $\Rightarrow \phi (1) = 2 \wedge \phi (d) = 0$ for d≠1 $\Rightarrow \delta (\phi ) = 1$ ⇒ Incorrect decision d=1.

We thus observe that for the learning rate parameter and the scenario considered, the agent makes correct decisions for all reward state-indicative rewards and makes incorrect decisions for both trials with a reward state-non-indicative reward. For an identical reward state and reward sequence, agent A3 with a medium learning rate thus makes more incorrect decisions than agent A3 with a small learning rate.

**Large learning rate parameter** η=2

Trial t=1, reward state-indicative reward $r_1 = (1,-1)^T$

$$\begin{aligned} \left( \begin{array}{r} \hat{\mu }^{o=3}_{{r_p}} \\ \hat{\mu }^{o=3}_{{r_n}} \\ \end{array}\right) _{1} =&\left( \begin{array}{r} 0 \\ 0 \end{array}\right) + 2 \left( \left( \begin{array}{r} 1 \\ -1 \\ \end{array}\right) - \left( \begin{array}{r} 0 \\ 0 \\ \end{array}\right) \right) \\ =&\left( \begin{array}{r} 0 \\ 0 \end{array}\right) + 2 \left( \begin{array}{r} 1 \\ -1 \\ \end{array}\right) \\ =&\left( \begin{array}{r} 0 \\ 0 \end{array}\right) + \left( \begin{array}{r} 2 \\ -2 \\ \end{array}\right) \\ =&\left( \begin{array}{r} 2 \\ -2 \\ \end{array}\right) \end{aligned}$$

$\Rightarrow \hat{\mu }_{r^p}^{o=3} \ge 0 \wedge \hat{\mu }_{r^n}^{o=3} < 0$ $\Rightarrow \phi (3) = 2 \wedge \phi (d) = 0$ for d≠3 $\Rightarrow \delta (\phi ) = 3$ ⇒ Correct decision d=3.

Trial t=2, reward state-non-indicative reward $r_2 = (1,1)^T$

$$\begin{aligned} \left( \begin{array}{r} \hat{\mu }^{o=3}_{{r_p}} \\ \hat{\mu }^{o=3}_{{r_n}} \\ \end{array}\right) _{2} =&\left( \begin{array}{r} 2 \\ -2 \end{array}\right) + 2 \left( \left( \begin{array}{r} 1 \\ 1 \\ \end{array}\right) - \left( \begin{array}{r} 2 \\ -2 \\ \end{array}\right) \right) \\ =&\left( \begin{array}{r} 2 \\ -2 \end{array}\right) + 2 \left( \begin{array}{r} -1 \\ 3 \\ \end{array}\right) \\ =&\left( \begin{array}{r} 2 \\ -2 \end{array}\right) + \left( \begin{array}{r} -2 \\ 6 \\ \end{array}\right) \\ =&\left( \begin{array}{r} 0 \\ 4 \\ \end{array}\right) \end{aligned}$$

$\Rightarrow \hat{\mu }_{r^p}^{o=3} \ge 0 \wedge \hat{\mu }_{r^n}^{o=3} \ge 0$ $\Rightarrow \phi (1) = 2 \wedge \phi (d) = 0$ for d≠1 $\Rightarrow \delta (\phi ) = 1$ ⇒ Incorrect decision d=1.

Trial t=3, reward state-indicative reward $r_3 = (1,-1)^T$

$$\begin{aligned} \left( \begin{array}{r} \hat{\mu }^{o=3}_{{r_p}} \\ \hat{\mu }^{o=3}_{{r_n}} \\ \end{array}\right) _{3} =&\left( \begin{array}{r} 0 \\ 4 \end{array}\right) + 2 \left( \left( \begin{array}{r} 1 \\ -1 \\ \end{array}\right) - \left( \begin{array}{r} 0 \\ 4 \\ \end{array}\right) \right) \\ =&\left( \begin{array}{r} 0 \\ 4 \end{array}\right) + 2 \left( \begin{array}{r} 1 \\ -5 \\ \end{array}\right) \\ =&\left( \begin{array}{r} 0 \\ 4 \end{array}\right) + \left( \begin{array}{r} 2 \\ -10 \\ \end{array}\right) \\ =&\left( \begin{array}{r} 2 \\ -6 \\ \end{array}\right) \end{aligned}$$

$\Rightarrow \hat{\mu }_{r^p}^{o=3} \ge 0 \wedge \hat{\mu }_{r^n}^{o=3} <0$ $\Rightarrow \phi (3) = 2 \wedge \phi (d) = 0$ for d≠3 $\Rightarrow \delta (\phi ) = 3$ ⇒ Correct decision d=3.

Trial t=4, reward state-indicative reward $r_4 = (1,-1)^T$

$$\begin{aligned} \left( \begin{array}{r} \hat{\mu }^{o=3}_{{r_p}} \\ \hat{\mu }^{o=3}_{{r_n}} \\ \end{array}\right) _{4} =&\left( \begin{array}{r} 2 \\ -6 \end{array}\right) + 2 \left( \left( \begin{array}{r} 1 \\ -1 \\ \end{array}\right) - \left( \begin{array}{r} 2 \\ -6 \end{array}\right) \right) \\ =&\left( \begin{array}{r} 2 \\ -6 \end{array}\right) + 2 \left( \begin{array}{r} -1 \\ 5 \\ \end{array}\right) \\ =&\left( \begin{array}{r} 2 \\ -6 \end{array}\right) + \left( \begin{array}{r} -2 \\ 10 \\ \end{array}\right) \\ =&\left( \begin{array}{r} 0\\ 4 \\ \end{array}\right) \end{aligned}$$

$\Rightarrow \hat{\mu }_{r^p}^{o=3} \ge 0 \wedge \hat{\mu }_{r^n}^{o=3} \ge 0$ $\Rightarrow \phi (1) = 2 \wedge \phi (d) = 0$ for d≠1 $\Rightarrow \delta (\phi ) = 1$ ⇒ Incorrect decision d=1.

Trial t=5, reward state-non-indicative reward $r_5 = (1,1)^T$

$$\begin{aligned} \left( \begin{array}{r} \hat{\mu }^{o=3}_{{r_p}} \\ \hat{\mu }^{o=3}_{{r_n}} \\ \end{array}\right) _{5} =&\left( \begin{array}{r} 0 \\ 4 \end{array}\right) + 2 \left( \left( \begin{array}{r} 1 \\ 1 \\ \end{array}\right) - \left( \begin{array}{r} 0 \\ 4 \end{array}\right) \right) \\ =&\left( \begin{array}{r} 0 \\ 4 \end{array}\right) + 2 \left( \begin{array}{r} 1 \\ -3 \\ \end{array}\right) \\ =&\left( \begin{array}{r} 0 \\ 4 \end{array}\right) + \left( \begin{array}{r} 2 \\ -6 \\ \end{array}\right) \\ =&\left( \begin{array}{r} 2\\ -2 \\ \end{array}\right) \end{aligned}$$

$\Rightarrow \hat{\mu }_{r^p}^{o=3} \ge 0 \wedge \hat{\mu }_{r^n}^{o=3} \le 0$ $\Rightarrow \phi (3) = 2 \wedge \phi (d) = 0$ for d≠3 $\Rightarrow \delta (\phi ) = 3$ ⇒ Correct decision d=3.

We thus observe that agent A3 with a large learning rate of η=2 makes incorrect decisions on both reward state-indicative and reward state-non-indicative trials, rendering this agent’s decision model essentially independent from the observed rewards. By considering three additional trials, all of which necessarily exhibit reward state-indicative rewards as the two reward state-non-indicative rewards have already been observed, we can demonstrate the deteriorating effect of the large learning rate for agent A3’s decision accuracy even further.

Trial t=6, reward state-indicative reward $r_6 = (1,-1)^T$

$$\begin{aligned} \left( \begin{array}{r} \hat{\mu }^{o=3}_{{r_p}} \\ \hat{\mu }^{o=3}_{{r_n}} \\ \end{array}\right) _{6} =&\left( \begin{array}{r} 2 \\ -2 \end{array}\right) + 2 \left( \left( \begin{array}{r} 1 \\ -1 \\ \end{array}\right) - \left( \begin{array}{r} 2 \\ -2 \end{array}\right) \right) \\ =&\left( \begin{array}{r} 2 \\ -2 \end{array}\right) + 2 \left( \begin{array}{r} -1 \\ 1 \\ \end{array}\right) \\ =&\left( \begin{array}{r} 2 \\ -2 \end{array}\right) + \left( \begin{array}{r} -2 \\ 2 \\ \end{array}\right) \\ =&\left( \begin{array}{r} 0\\ 0 \\ \end{array}\right) \end{aligned}$$

$\Rightarrow \hat{\mu }_{r^p}^{o=3} \ge 0 \wedge \hat{\mu }_{r^n}^{o=3} \ge 0$ $\Rightarrow \phi (1) = 2 \wedge \phi (d) = 0$ for d≠1 $\Rightarrow \delta (\phi ) = 1$ ⇒ Incorrect decision d=1.

Trial t=7, reward state-indicative reward $r_7 = (1,-1)^T$

$$\begin{aligned} \left( \begin{array}{r} \hat{\mu }^{o=3}_{{r_p}} \\ \hat{\mu }^{o=3}_{{r_n}} \\ \end{array}\right) _{7} =&\left( \begin{array}{r} 0 \\ 0 \end{array}\right) + 2 \left( \left( \begin{array}{r} 1 \\ -1 \\ \end{array}\right) - \left( \begin{array}{r} 0 \\ 0 \\ \end{array}\right) \right) \\ =&\left( \begin{array}{r} 0 \\ 0 \end{array}\right) + 2 \left( \begin{array}{r} 1 \\ -1 \\ \end{array}\right) \\ =&\left( \begin{array}{r} 0 \\ 0 \end{array}\right) + \left( \begin{array}{r} 2 \\ -2 \\ \end{array}\right) \\ =&\left( \begin{array}{r} 2 \\ -2 \\ \end{array}\right) \end{aligned}$$

$\Rightarrow \hat{\mu }_{r^p}^{o=3} \ge 0 \wedge \hat{\mu }_{r^n}^{o=3} < 0$ $\Rightarrow \phi (3) = 2 \wedge \phi (d) = 0$ for d≠3 $\Rightarrow \delta (\phi ) = 3$ ⇒ Correct decision d=3.

Trial t=8, reward state-indicative reward $r_8 = (1,-1)^T$

$$\begin{aligned} \left( \begin{array}{r} \hat{\mu }^{o=3}_{{r_p}} \\ \hat{\mu }^{o=3}_{{r_n}} \\ \end{array}\right) _{8} =&\left( \begin{array}{r} 2 \\ -2 \end{array}\right) + 2 \left( \left( \begin{array}{r} 1 \\ -1 \\ \end{array}\right) - \left( \begin{array}{r} 2 \\ -2 \end{array}\right) \right) \\ =&\left( \begin{array}{r} 2 \\ -2 \end{array}\right) + 2 \left( \begin{array}{r} -1 \\ 1 \\ \end{array}\right) \\ =&\left( \begin{array}{r} 2 \\ -2 \end{array}\right) + \left( \begin{array}{r} -2 \\ 2 \\ \end{array}\right) \\ =&\left( \begin{array}{r} 0\\ 0 \\ \end{array}\right) \end{aligned}$$

$\Rightarrow \hat{\mu }_{r^p}^{o=3} \ge 0 \wedge \hat{\mu }_{r^n}^{o=3} \ge 0$ $\Rightarrow \phi (1) = 2 \wedge \phi (d) = 0$ for d≠1 $\Rightarrow \delta (\phi ) = 1$ ⇒ Incorrect decision d=1.

### E.2 Agent A4

To illustrate the effect of an increase of the positive learning rate parameter from $\eta _p = 1.6$ to $\eta _p = 2.0$ on the decision accuracy of agent A4, we consider the expected reward value estimate updates and the subsequent decisions for an exemplary sequence of trials for reward state s=0 (low positive reward, low negative reward) and a negative learning rate parameter of $\eta _n = 0.01$. As in E.1 Agent A3, it suffices to consider the respective visual search field pattern observation-dependent components of the expected reward value estimate vector. As shown in the following, it further suffices to consider two trials with the reward state-indicative reward $r = (-1,+1)^T$.

**Positive learning rate parameter** $\eta _p = 1.6$

Trial t=1, reward state-indicative reward $r_1 = (-1,1)^T$

$$\begin{aligned} \left( \begin{array}{r} \hat{\mu }^{o=1}_{{r_p}} \\ \hat{\mu }^{o=1}_{{r_n}} \\ \end{array}\right) _{1} =&\left( \begin{array}{r} 0.000 \\ 0.000 \end{array}\right) + \left( \begin{array}{r} 1.600 \\ 0.010 \end{array}\right) \circ \left( \left( \begin{array}{r} -1 \\ 1 \\ \end{array}\right) - \left( \begin{array}{r} 0.000 \\ 0.000 \\ \end{array}\right) \right) \\ =&\left( \begin{array}{r} 0.000 \\ 0.000 \\ \end{array}\right) + \left( \begin{array}{r} 1.600 \\ 0.010 \end{array}\right) \circ \left( \begin{array}{r} -1.000 \\ 1.000 \\ \end{array}\right) \\ =&\left( \begin{array}{r} 0.000 \\ 0.000 \\ \end{array}\right) + \left( \begin{array}{r} -1.600 \\ 1.000 \\ \end{array}\right) \nonumber \\ =&\left( \begin{array}{r} -1.600 \\ 1.000 \\ \end{array}\right) \end{aligned}$$

$\Rightarrow \hat{\mu }_{r^p}^{o=1} < 0 \wedge \hat{\mu }_{r^n}^{o=1} \ge 0$ $\Rightarrow \phi (0) = 2 \wedge \phi (d) = 0$ for d≠0 $\Rightarrow \delta (\phi ) = 0$ ⇒ Correct decision d=0.

Trial t=2, reward state-indicative reward $r_2 = (-1,1)^T$

$$\begin{aligned} \left( \begin{array}{r} \hat{\mu }^{o=1}_{{r_p}} \\ \hat{\mu }^{o=1}_{{r_n}} \\ \end{array}\right) _{2} =&\left( \begin{array}{r} -1.600 \\ 0.010 \end{array}\right) + \left( \begin{array}{r} 1.600 \\ 0.010 \end{array}\right) \circ \left( \left( \begin{array}{r} -1 \\ 1 \\ \end{array}\right) - \left( \begin{array}{r} -1.600 \\ 0.010 \end{array}\right) \right) \nonumber \\ =&\left( \begin{array}{r} -1.600 \\ 0.010 \end{array}\right) + \left( \begin{array}{r} 1.600 \\ 0.010 \end{array}\right) \circ \left( \begin{array}{r} 0.600 \\ 0.990 \\ \end{array}\right) \\ =&\left( \begin{array}{r} -1.600 \\ 0.010 \end{array}\right) + \left( \begin{array}{r} 0.960 \\ 0.010 \\ \end{array}\right) \nonumber \\ =&\left( \begin{array}{r} -0.640\\ 0.020\\ \end{array}\right) \end{aligned}$$

$\Rightarrow \hat{\mu }_{r^p}^{o=1} < 0 \wedge \hat{\mu }_{r^n}^{o=1} \ge 0$ $\Rightarrow \phi (0) = 2 \wedge \phi (d) = 0$ for d≠0 $\Rightarrow \delta (\phi ) = 0$ ⇒ Correct decision d=0.

**Positive learning rate parameter** $\eta _p = 2$

Trial t=1, reward state-indicative reward $r_1 = (-1,1)^T$

$$\begin{aligned} \left( \begin{array}{r} \hat{\mu }^{o=1}_{{r_p}} \\ \hat{\mu }^{o=1}_{{r_n}} \\ \end{array}\right) _{1} =&\left( \begin{array}{r} 0.000 \\ 0.000 \end{array}\right) + \left( \begin{array}{r} 2.000 \\ 0.010 \end{array}\right) \circ \left( \left( \begin{array}{r} -1 \\ 1 \\ \end{array}\right) - \left( \begin{array}{r} 0.000 \\ 0.000 \\ \end{array}\right) \right) \nonumber \\ =&\left( \begin{array}{r} 0.000 \\ 0.000 \\ \end{array}\right) + \left( \begin{array}{r} 2.000 \\ 0.010 \end{array}\right) \circ \left( \begin{array}{r} -1.000 \\ 1.000 \\ \end{array}\right) \\ =&\left( \begin{array}{r} 0.000 \\ 0.000 \\ \end{array}\right) + \left( \begin{array}{r} -2.000 \\ 0.010 \end{array}\right) \nonumber \\ =&\left( \begin{array}{r} -2.000 \\ 0.010 \end{array}\right) \end{aligned}$$

$\Rightarrow \hat{\mu }_{r^p}^{o=1} < 0 \wedge \hat{\mu }_{r^n}^{o=1} \ge 0$ $\Rightarrow \phi (0) = 2 \wedge \phi (d) = 0$ for d≠0 $\Rightarrow \delta (\phi ) = 0$ ⇒ Correct decision d=0.

Trial t=2, reward state-indicative reward $r_2 = (-1,1)^T$

$$\begin{aligned} \left( \begin{array}{r} \hat{\mu }^{o=1}_{{r_p}} \\ \hat{\mu }^{o=1}_{{r_n}} \\ \end{array}\right) _{2} =&\left( \begin{array}{r} -2.000 \\ 0.010 \end{array}\right) + \left( \begin{array}{r} 2.000 \\ 0.010 \end{array}\right) \circ \left( \left( \begin{array}{r} -1 \\ 1 \\ \end{array}\right) - \left( \begin{array}{r} -2.000 \\ 0.010 \end{array}\right) \right) \nonumber \\ =&\left( \begin{array}{r} -2.000 \\ 0.010 \end{array}\right) + \left( \begin{array}{r} 2.000 \\ 0.010 \end{array}\right) \circ \left( \begin{array}{r} 1.000 \\ 0.990 \\ \end{array}\right) \\ =&\left( \begin{array}{r} -2.000 \\ 0.010 \end{array}\right) + \left( \begin{array}{r} 2.000 \\ 0.010 \\ \end{array}\right) \nonumber \\ =&\left( \begin{array}{r} 0.000\\ 0.020\\ \end{array}\right) \end{aligned}$$

$\Rightarrow \hat{\mu }_{r^p}^{o=1} \ge 0 \wedge \hat{\mu }_{r^n}^{o=1} \ge 0$ $\Rightarrow \phi (1) = 2 \wedge \phi (d) = 0$ for d≠1 $\Rightarrow \delta (\phi ) = 1$ ⇒ Incorrect decision d=1.

We thus observe that while agent A4 makes correct decisions in the scenario considered if the positive learning rate parameter is set to $\eta _p = 1.6$, for a positive learning rate parameter set to $\eta _p = 2.0$, the agent makes incorrect decisions on trials with reward state-indicative rewards.

### E.3 Agent A5

To illustrate the differential effect of the prior expectation bias parameter for low and high negative reward states at a small learning rate parameter, we consider the expected reward value estimate updates and the subsequent decisions for a positive prior expectation bias parameter value of π=0.2 and an exemplary sequence comprising three trials for the reward states s=1 (high positive reward, low negative reward) and s=3 (high positive reward, high negative reward). As above, it suffices to consider the respective visual search field pattern observation-dependent components of the expected reward value estimate vector. Similarly, for negative values of π the reported effects of π on the reward state-specific decision-making behavior reverse.

**Prior expectation bias parameter** π=0.2

Trial t=1, reward state-indicative reward $r_1 = (1,1)^T$

$$\begin{aligned} \left( \begin{array}{r} \hat{\mu }^{o=1}_{{r_p}} \\ \hat{\mu }^{o=1}_{{r_n}} \\ \end{array}\right) _{1} =&\left( \begin{array}{r} 0.000 \\ 0.200 \end{array}\right) + 0.01 \left( \left( \begin{array}{r} 1 \\ 1 \\ \end{array}\right) - \left( \begin{array}{r} 0.000 \\ 0.200 \end{array}\right) \right) \\ =&\left( \begin{array}{r} 0.000 \\ 0.200 \end{array}\right) + 0.01 \left( \begin{array}{r} 1.000 \\ 0.800 \\ \end{array}\right) \\ =&\left( \begin{array}{r} 0.000 \\ 0.000 \\ \end{array}\right) + \left( \begin{array}{r} 0.010 \\ 0.008 \\ \end{array}\right) \\ =&\left( \begin{array}{r} 0.010 \\ 0.208 \\ \end{array}\right) \end{aligned}$$

$\Rightarrow \hat{\mu }_{r^p}^{o=1} \ge 0 \wedge \hat{\mu }_{r^n}^{o=1} \ge 0$ $\Rightarrow \phi (1) = 2 \wedge \phi (d) = 0$ for d≠1 $\Rightarrow \delta (\phi ) = 1$ ⇒ Correct decision d=1.

Trial t=2, reward state-non-indicative reward $r_2 = (1,-1)^T$

$$\begin{aligned} \left( \begin{array}{r} \hat{\mu }^{o=1}_{{r_p}} \\ \hat{\mu }^{o=1}_{{r_n}} \\ \end{array}\right) _{2} =&\left( \begin{array}{r} 0.010 \\ 0.208 \\ \end{array}\right) + 0.01 \left( \left( \begin{array}{r} 1 \\ -1 \\ \end{array}\right) - \left( \begin{array}{r} 0.010 \\ 0.208 \\ \end{array}\right) \right) \\ =&\left( \begin{array}{r} 0.010 \\ 0.208 \\ \end{array}\right) + 0.01 \left( \begin{array}{r} 0.990 \\ -1.208 \\ \end{array}\right) \\ =&\left( \begin{array}{r} 0.010 \\ 0.208 \\ \end{array}\right) + \left( \begin{array}{r} 0.010 \\ -0.012 \\ \end{array}\right) \\ =&\left( \begin{array}{r} 0.020 \\ 0.196 \\ \end{array}\right) \end{aligned}$$

$\Rightarrow \hat{\mu }_{r^p}^{o=1} \ge 0 \wedge \hat{\mu }_{r^n}^{o=1} \ge 0$ $\Rightarrow \phi (1) = 2 \wedge \phi (d) = 0$ for d≠1 $\Rightarrow \delta (\phi ) = 1$ ⇒ Correct decision d=1.

Trial t=3, reward state-indicative reward $r_3 = (1,1)^T$

$$\begin{aligned} \left( \begin{array}{r} \hat{\mu }^{o=1}_{{r_p}} \\ \hat{\mu }^{o=1}_{{r_n}} \\ \end{array}\right) _{3} =&\left( \begin{array}{r} 0.020 \\ 0.196 \\ \end{array}\right) + 0.01 \left( \left( \begin{array}{r} 1 \\ 1 \\ \end{array}\right) - \left( \begin{array}{r} 0.020 \\ 0.196 \\ \end{array}\right) \right) \\ =&\left( \begin{array}{r} 0.020 \\ 0.196 \\ \end{array}\right) + 0.01 \left( \begin{array}{r} 0.980 \\ 0.804 \\ \end{array}\right) \\ =&\left( \begin{array}{r} 0.020 \\ 0.196 \\ \end{array}\right) + \left( \begin{array}{r} 0.010 \\ 0.008 \\ \end{array}\right) \\ =&\left( \begin{array}{r} 0.030 \\ 0.204 \\ \end{array}\right) \end{aligned}$$

$\Rightarrow \hat{\mu }_{r^p}^{o=1} \ge 0 \wedge \hat{\mu }_{r^n}^{o=1} \ge 0$ $\Rightarrow \phi (1) = 2 \wedge \phi (d) = 0$ for d≠1 $\Rightarrow \delta (\phi ) = 1$ ⇒ Correct decision d=1.

We thus observe that with a positive prior expectation bias parameter of π=0.2, the agent makes no incorrect decisions in the scenario of a low negative reward state.

**Prior expectation bias parameter** π=0.2

Trial t=1, reward state-indicative reward $r_1 = (1,-1)^T$

$$\begin{aligned} \left( \begin{array}{r} \hat{\mu }^{o=3}_{{r_p}} \\ \hat{\mu }^{o=3}_{{r_n}} \\ \end{array}\right) _{1} =&\left( \begin{array}{r} 0.000 \\ 0.200 \end{array}\right) + 0.01 \left( \left( \begin{array}{r} 1 \\ -1 \\ \end{array}\right) - \left( \begin{array}{r} 0.000 \\ 0.200 \end{array}\right) \right) \\ =&\left( \begin{array}{r} 0.000 \\ 0.200 \end{array}\right) + 0.01 \left( \begin{array}{r} 1.000 \\ -1.200 \\ \end{array}\right) \\ =&\left( \begin{array}{r} 0.000 \\ 0.200 \\ \end{array}\right) + \left( \begin{array}{r} 0.010 \\ -0.012 \\ \end{array}\right) \\ =&\left( \begin{array}{r} 0.010 \\ 0.188 \\ \end{array}\right) \end{aligned}$$

$\Rightarrow \hat{\mu }_{r^p}^{o=3} \ge 0 \wedge \hat{\mu }_{r^n}^{o=3}\ge 0$ $\Rightarrow \phi (1) = 2 \wedge \phi (d) = 0$ for d≠1 $\Rightarrow \delta (\phi ) = 1$ ⇒ Incorrect decision d=1.

Trial t=2, reward state-non-indicative reward $r_2 = (1,1)^T$

$$\begin{aligned} \left( \begin{array}{r} \hat{\mu }^{o=3}_{{r_p}} \\ \hat{\mu }^{o=3}_{{r_n}} \\ \end{array}\right) _{2} =&\left( \begin{array}{r} 0.010 \\ 0.188 \\ \end{array}\right) + 0.01 \left( \left( \begin{array}{r} 1 \\ 1 \\ \end{array}\right) - \left( \begin{array}{r} 0.010 \\ 0.188 \\ \end{array}\right) \right) \\ =&\left( \begin{array}{r} 0.010 \\ 0.188 \\ \end{array}\right) + 0.01 \left( \begin{array}{r} 0.990 \\ 0.812 \\ \end{array}\right) \\ =&\left( \begin{array}{r} 0.010 \\ 0.188 \\ \end{array}\right) + \left( \begin{array}{r} 0.010 \\ 0.008 \\ \end{array}\right) \\ =&\left( \begin{array}{r} 0.020 \\ 0.196 \\ \end{array}\right) \end{aligned}$$

$\Rightarrow \hat{\mu }_{r^p}^{o=3} \ge 0 \wedge \hat{\mu }_{r^n}^{o=3}\ge 0$ $\Rightarrow \phi (1) = 2 \wedge \phi (d) = 0$ for d≠1 $\Rightarrow \delta (\phi ) = 1$ ⇒ Incorrect decision d=1.

Trial t=3, reward state-indicative reward $r_3 = (1,-1)^T$

$$\begin{aligned} \left( \begin{array}{r} \hat{\mu }^{o=3}_{{r_p}} \\ \hat{\mu }^{o=3}_{{r_n}} \\ \end{array}\right) _{3} =&\left( \begin{array}{r} 0.020 \\ 0.196 \\ \end{array}\right) + 0.01 \left( \left( \begin{array}{r} 1 \\ -1 \\ \end{array}\right) - \left( \begin{array}{r} 0.020 \\ 0.196 \\ \end{array}\right) \right) \\ =&\left( \begin{array}{r} 0.020 \\ 0.196 \\ \end{array}\right) + 0.01 \left( \begin{array}{r} 0.980 \\ -1.196 \\ \end{array}\right) \\ =&\left( \begin{array}{r} 0.020 \\ 0.196 \\ \end{array}\right) + \left( \begin{array}{r} 0.010 \\ -0.012 \\ \end{array}\right) \\ =&\left( \begin{array}{r} 0.030 \\ 0.184 \\ \end{array}\right) \end{aligned}$$

$\Rightarrow \hat{\mu }_{r^p}^{o=3} \ge 0 \wedge \hat{\mu }_{r^n}^{o=3}\ge 0$ $\Rightarrow \phi (1) = 2 \wedge \phi (d) = 0$ for d≠1 $\Rightarrow \delta (\phi ) = 1$ ⇒ Incorrect decision d=1.

We thus observe that with a positive prior expectation bias parameter of π=0.2, the agent makes incorrect decisions in the scenario of a high negative reward state.

To replicate the results of this Appendix, please refer to *abm_appendix_e.py*.

## Appendix F: τ-Non-identifiability

As detailed in the main text, due to agent’s A0 identical decision values, a parameter-dependent embedding of the control agent’s decision value function of the form (26) was neither required nor possible, as the embedding of the decision function values of agent A0 in such a model would result in a uniform probability distribution for any value of the respective parameter and would thus be identical to the agent’s decision distribution (cf. (6)). We show this by using the concept of parameter identifiability in probabilistic models. To this end, we follow Casella and Berger (2002, p. 523) and call the τ parameter of a model of the form of (26) *identifiable*, if for all $\tau _1,\tau _2>0$,

F7 $$\begin{aligned} \tau _1 \ne \tau _2 \Rightarrow p^{\tau _1}(a_t) \ne p^{\tau _2}(a_t). \end{aligned}$$

Next, we consider embedding the decision value function of agent A0 in (26), such that $v(a) = v(d):= \frac{1}{4}$ for a=d for two arbitrary, but distinct parameter values $\tau _1 \ne \tau _2$. Then, for all a∈A,

F8 $$\begin{aligned} p^{\tau _1}(a_t = a)&= \frac{\exp (\tau _1^{-1}v(a))}{\sum _{a^{\prime } \in A}\exp (\tau _1^{-1}v(a^{\prime }))}= \frac{\exp (\tau _1^{-1}v(d))}{4\exp (\tau _1^{-1}v(d))}\nonumber \\&= \frac{1}{4}= v(a)= v(d), \end{aligned}$$

and similarly, for all a∈A,

F9 $$\begin{aligned} p^{\tau _2}(a_t = a)&= \frac{\exp (\tau _2^{-1}v(a))}{\sum _{a^{\prime } \in A}\exp (\tau _2^{-1}v(a^{\prime }))}= \frac{\exp (\tau _2^{-1}v(d))}{4\exp (\tau _2^{-1}v(d))}\nonumber \\&= \frac{1}{4}= v(a)= v(d). \end{aligned}$$

Thus, because $v(0) = v(1) = v(2) = v(3):= \frac{1}{4}$, it does not follow from $\tau _1 \ne \tau _2$ that the action distributions $p^{\tau _1}(a_t)$ and $p^{\tau _2}(a_t)$ are distinct and, in the scenario of all identical action values *v*(*a*) for a∈A, τ is thus not identifiable.
